# The impact of adverse childhood experiences on the development of multimorbidity in older adults in Nigeria

**DOI:** 10.1371/journal.pone.0358944

**Published:** 2026-09-25

**Authors:** Abdulsalam Ahmed, Hafiz T. A. Khan, Nestor Asiamah

**Affiliations:** 1 Department of Public Health/Community Health, Newgate University Minna, Niger state Nigeria & Ranc Orbit International Ltd/Gte, Minna, Niger State, Nigeria; 2 College of Nursing, Midwifery, and Healthcare, University of West London, London, United Kingdom; 3 Oxford Institute of Population Ageing, University of Oxford, Oxford, United Kingdom; 4 School of Health and Social Care, University of Essex, Colchester, Essex, United Kingdom; Anthropological Survey of India, Government of India, INDIA

## Abstract

**Background:**

Adverse Childhood Experiences (ACEs) are established life-course determinants of adult health, yet evidence linking ACEs to multimorbidity in sub-Saharan Africa remains limited. Nigeria is undergoing rapid demographic and epidemiological transitions characterized by population ageing and a rising burden of non-communicable diseases (NCDs). This study examined whether cumulative ACE exposure predicts multimorbidity among older adults in North-Central Nigeria.

**Methods:**

A cross-sectional study was conducted among 734 adults aged ≥60 years in Niger State, Nigeria. ACE exposure before age 18 was assessed using the Adverse Childhood Experiences International Questionnaire (ACE-IQ) across 13 domains. Multimorbidity was defined as the presence of two or more chronic conditions (NICE criteria) from a list of 24 conditions and analyzed both as a count and dichotomized (≥3 vs. < 3 conditions). Descriptive statistics summarized participant characteristics. Chi-square tests examined bivariate associations. Hierarchical logistic regression models estimated adjusted odds ratios (ORs) and 95% confidence intervals (CIs) to determine independent predictors of multimorbidity.

**Results:**

High cumulative ACE exposure was observed, with 68.4% reporting ≥4 ACEs. Multimorbidity was common: 38.4% reported three or more chronic conditions. ACE exposure was strongly associated with multimorbidity (χ² = 116.07, p < 0.001). In unadjusted analysis, participants reporting ≥4 ACEs had significantly higher odds of multimorbidity (OR = 67.14; 95% CI: 9.25–487.04). After adjusting for socio-demographic covariates, cumulative ACE exposure remained an independent predictor (OR = 15.58; 95% CI: 1.33–182.21). Advanced age (≥80 years) and living in three-generation households were also independently associated with multimorbidity.

**Conclusions:**

Cumulative childhood adversity significantly predicts multimorbidity among older Nigerian adults, demonstrating a clear dose–response relationship. These findings underscore the importance of life-course approaches and trauma-informed strategies in public health policy. Early identification and prevention of childhood adversity may contribute to reducing the long-term burden of multimorbidity in low- and middle-income settings.

## Background

Adverse Childhood Experiences (ACEs) are increasingly recognized as critical early-life determinants of health across the life course. ACEs refer to potentially traumatic events occurring before the age of 18 years that threaten a child’s physical or psychological well-being. These include child maltreatment (physical, emotional, or sexual abuse) and household dysfunction such as exposure to domestic violence, parental separation, substance misuse, mental illness, or incarceration within the household [1]. Since the landmark ACE Study, evidence has consistently demonstrated a graded relationship between cumulative childhood adversity and adverse health outcomes in adulthood [2]. Recent systematic reviews have reinforced this, showing that individuals with multiple ACEs are at significantly higher risk of chronic diseases in later life [3].

A substantial body of epidemiological research shows that individuals exposed to ACEs are at increased risk of chronic diseases, including hypertension, diabetes mellitus, cardiovascular diseases, and depression [2,3]. ACE exposure is also associated with higher likelihood of engaging in health-risk behaviors—such as tobacco use, harmful alcohol consumption, physical inactivity, and risky sexual behavior—which further elevate the risk of non-communicable diseases (NCDs) and premature mortality [3,5]. Importantly, ACEs frequently co-occur; exposure to one adverse event increases the likelihood of exposure to others, leading to cumulative biological and psychosocial stress [3,4]. Neurobiological evidence suggests that early-life toxic stress can result in long-term dysregulation of neuroendocrine, immune, and metabolic systems, thereby increasing vulnerability to chronic disease across the lifespan [5].

These long-term consequences are particularly relevant within the framework of multimorbidity, defined as the coexistence of two or more chronic conditions in an individual [6]. Globally, multimorbidity has emerged as a major public health challenge driven by population aging and epidemiological transition [6]. It is associated with increased healthcare utilization, reduced quality of life, polypharmacy, functional decline, and higher mortality [7]. Clinical management is complex because healthcare systems remain largely structured around single-disease models, and applying disease-specific guidelines to individuals with multiple conditions often results in treatment burden and fragmented care [8].

Understanding multimorbidity requires a life-course perspective that goes beyond single-disease etiology. Evidence suggests that the accumulation of chronic conditions is shaped by genetic predisposition, environmental exposures, behavioral risk factors, and social determinants of health [9]. From a public health standpoint, identifying early-life modifiable risk factors is crucial for prevention. Risk factors for NCDs accumulate across the lifespan, and early intervention during critical developmental windows may delay or compress the onset of chronic diseases in later life [10]. ACEs represent one such early-life exposure that may contribute to disease accumulation and multimorbidity in adulthood [3,4].

In low- and middle-income countries (LMICs), including Nigeria, the relevance of this life-course approach is particularly pronounced. Nigeria is undergoing demographic and epidemiological transitions characterized by a growing population of older adults and a rising burden of NCDs(11). NCDs account for approximately one-third of adult mortality in Nigeria, with cardiovascular diseases and diabetes among the leading contributors [11]. As survival improves and life expectancy increases, the prevalence of multimorbidity is also rising. However, the healthcare system remains largely oriented toward acute and single-disease management, with limited geriatric specialization and insufficient integration of preventive life-course strategies.

Despite evidence that childhood adversity is prevalent in many LMIC contexts, including poverty, exposure to violence, family instability, and limited social protection, there is limited empirical research examining the contribution of ACEs to multimorbidity among older adults in Nigeria. Most existing evidence on ACEs and chronic disease originates from high-income settings, limiting contextual applicability to African populations [3,4].

Previous studies have consistently shown that cumulative exposure to adverse childhood experiences (ACEs) is associated with an increased risk of chronic disease and multimorbidity in later life [2–4]. Evidence indicates a dose–response relationship, where individuals with a higher number of ACEs are more likely to develop multiple chronic conditions compared with those with few or no ACEs. These associations persist even after accounting for sociodemographic factors such as age, gender, education, and family structure. Earlier research underscores that ACEs contribute to multimorbidity through both behavioral and biological pathways, highlighting the importance of life-course approaches and early preventive strategies to mitigate long-term health risks [2–4]. Identifying ACEs as an upstream determinant of multimorbidity has important implications for policy, prevention, and clinical care. Early interventions targeting children at risk of adversity may reduce the future burden of chronic disease and multimorbidity, particularly in LMICs where healthcare systems face resource constraints [3,4,9].

Biological and neurodevelopmental evidence provides plausible mechanisms linking adverse childhood experiences (ACEs) to later-life health outcomes. Research indicates that early adversity can induce lasting alterations in brain structure and function, with consequences for physical and mental health across the lifespan [12]. These neurobiological disruptions may persist into adulthood, increasing vulnerability to chronic disease development and progression [12]. Although the ACE framework has been widely used to retrospectively assess childhood adversity and its long-term health effects [13], comparatively less attention has been paid to broader measures of mental health, well-being, and modifiable life-course risk factors. Importantly, early life represents a critical window for preventive intervention [2].

Against the backdrop of a rapidly ageing population and rising multimorbidity in Nigeria and globally, health systems, often constrained by limited geriatric expertise, face increasing challenges [14]. Identifying modifiable early-life determinants such as ACEs may therefore inform prevention strategies aimed at delaying or compressing multimorbidity. Extending previous research, this study examined whether cumulative ACE exposure predicts multimorbidity, assessing the graded (dose–response) relationship between ACE burden and multimorbidity among older adults with established chronic conditions. By doing so, it contributes to the growing evidence base on the long-term health consequences of childhood adversity in north-central Nigeria.

## Methods

### Study design and ethical approval

A minimum sample size of 800 was calculated using the standard formula for large populations (N > 10,000) with a 5% margin of error. Based on hospital records, approximately 105,000 patients aged ≥60 years constituted the sampling frame. The initial sample size estimate was ~ 400, which was doubled to 800 to improve precision and account for potential non-response. A purposive selection was used to identify four high-volume public secondary hospitals (Bida, Kontagora, Minna, and Suleja) across Niger State. The study adopted a cross-sectional design targeting older adults (≥60 years) with multimorbidity (≥2 chronic conditions) attending outpatient clinics. Systematic random sampling was used to recruit eligible participants during routine clinic attendance. Data were collected from October 2021 to February 2022 using a structured, pre-tested questionnaire administered via face-to-face interviews. The instrument was deployed through the JISC online platform (University of West London). Participants were eligible if they were aged ≥60 years, had multimorbidity, attended selected outpatient departments during the study period, and could provide informed responses. Written informed consent was obtained from all participants. Data confidentiality was maintained, and participants retained the right to withdraw at any time. Interviews were conducted in English or Hausa according to participant preference. This clinic-based geriatric study was conducted as part of a larger investigation, with detailed methodology reported in previous publications [15–17].

Ethical approval was obtained from the College of Nursing, Midwifery, and Healthcare Research Ethics Panel, University of West London (Approval No. 1055), and authorization to collect data was granted by the Research, Ethics, and Publication Committee (REPC) of the Hospitals Management Board, Minna, Niger State, Nigeria. Written informed consent was obtained from all participants before partaking.

### Measurement of variables

#### Socio-demographic characteristics.

Participant demographics collected included age, sex, ethnicity, marital status, family structure, education level, occupation, and monthly household income. Age was treated as a continuous variable and later grouped into 10-year intervals. Marital status was classified as never married, currently married, divorced, separated, or widowed. Education level was categorized from illiterate to postgraduate. Occupation was classified as government employee, business owner, family business worker, company staff/worker, dependent, retired, or other. Family structure was grouped as nuclear, three-generation, or extended family.

#### Adverse childhood experiences (ACEs).

ACE exposure was measured using the Adverse Childhood Experiences International Questionnaire (ACE-IQ), a validated instrument widely used in large-scale studies of childhood adversity. Participants reported experiences before age 18 across 13 domains: physical abuse, emotional abuse, sexual abuse, household alcohol or drug abuse, incarcerated household member, living with someone chronically depressed, mentally ill, institutionalized, or suicidal, household violence, parental separation/divorce, emotional neglect, physical neglect, bullying, community violence, and collective violence. Responses were dichotomized (yes = 1, no = 0), summed to generate a cumulative ACE score (0–13), and categorized into five groups: 0, 1, 2, 3, and ≥4.

#### Multimorbidity.

Multimorbidity was defined according to NICE guidelines as the presence of two or more chronic conditions. A total of 24 conditions were assessed, including hypertension, diabetes, peptic ulcer, arthritis, heart failure, stroke, angina, COPD, chronic liver disease, obesity, depression, asthma, cataract, chronic renal failure, osteoporosis, glaucoma, tuberculosis, cancer, Alzheimer’s disease, dementia, and other mental or emotional illnesses. Self-reported diagnoses were cross-checked against medical records when available. Multimorbidity was analyzed both as a simple count of chronic conditions and categorized as 2, 3, 4, or ≥5 conditions.

#### Confounding factors.

Potential confounders included age, sex, marital status, education, occupation, family structure, ethnicity, and income. These variables were included in multivariable analyses to adjust for their potential influence on multimorbidity.

### Statistical analysis

Data were collated, coded, and entered using the JISC online platform, then exported to IBM SPSS version 27. Descriptive statistics summarized participant characteristics, ACE exposure, and multimorbidity status. Categorical variables were expressed as frequencies and percentages.

Bivariate associations between multimorbidity and selected variables were assessed using Chi-square (χ²) tests, with Fisher’s exact test applied where expected counts were <5. Variables examined included age, gender, marital status, family structure, education, occupation, income, ethnicity, and ACE score. Statistical significance was set at p < 0.05.

Hierarchical logistic regression was conducted to identify independent predictors of multimorbidity (≥3 chronic conditions). Two models were constructed:

- Model 1 included only ACE variables to examine unadjusted associations.
- Model 2 included ACEs and socio-demographic covariates (age, sex, marital status, education, occupation, income, family structure, and ethnicity) to adjust for confounding.

ACE scores were treated as categorical variables (0, 1, 2, 3, ≥ 4). Odds ratios (ORs) with 95% confidence intervals (CIs) were calculated. Model fit was evaluated using the Hosmer–Lemeshow goodness-of-fit test, and multicollinearity was assessed using variance inflation factors (VIFs), with VIF < 5 considered acceptable.

Model adequacy was assessed using the Hosmer–Lemeshow goodness-of-fit test, while multicollinearity among independent variables was evaluated using Variance Inflation Factors (VIFs). All VIF values were within acceptable limits, indicating no evidence of significant collinearity.

## Results

### Participant characteristics

A total of 800 older adults were initially recruited for this study. Of these, 734 (91.75%) completed all required study procedures and were included in the final analysis. The majority were female (59.1%) and aged 60–69 years (72.1%). Most were currently married (65.8%) and lived in extended families (60.4%). Regarding education, 62.9% were illiterate, and only 2.2% had postgraduate qualifications. Common occupations included business ownership (38.1%) and dependence on family support (29.2%). Participants were predominantly of Nupe (27.8%) and Gwarri (26.3%) ethnicity. Household income was generally low, with 65% earning ≤15,000 Naira monthly. High cumulative ACE exposure was reported, with 68.4% experiencing four or more ACEs. Multimorbidity was common, with 38.4% reporting three or more chronic conditions (Tables 1, 2 and 3).

**Table 1:** Socio-demographic characteristics of study participants (N = 734).

Variables	n	%
Gender		1
Male	300	40.9
Female	434	59.1
Age		
60-64	267	35.7
65-69	123	36.4
70-74	262	16.8
75-79	29	4.0
80 and greater	53	7.2
Marital status		
Never married	11	1.5
Currently married	483	65.8
Divorced	21	2.9
Separated	19	2.6
Widow/er	200	27.2
Family structure		
Nuclear Family	140	19.1
Three Generation Family	150	20.5
Extended Family	442	60.4
Education level		
Illiterate	462	62.9
Can read and write	35	4.8
Primary school level	74	10.1
secondary school	64	8.7
Tertiary school	83	11.3
Post-graduate	16	2.2
Occupation		
Government staff	36	4.9
Own business	280	38.1
Involve in the family business	36	4.9
Company staff/ worker	30	4.1
Dependent	214	29.2
Retired	128	17.4
Others (specify)	10	1.4
Ethnicity		
Gwarri	193	26.3
Hausa	174	23.7
Nupe	204	27.8
Others	163	22.2
Level of income (Naira)		
0-15k	477	65.0
16k-30k	124	16.9
31k-45k	30	4.1
46k-60k	27	3.7
greater than 60	76	10.4

**Table 2:** Frequency and percentage distribution of ACE exposure and multimorbidity.

ACE/Multimorbidity	Cases (n)	%
**ACE**		
0	6	0.8
1	44	6.0
2	65	8.7
3	117	15.9
4+	502	68.4
**Multimorbidity**		
2	452	61.6
3	230	31.3
4	38	5.2
5+	14	1.9

**Table 3:** Bivariate associations between multimorbidity and selected independent variables.

Variables	<3 diseases n (%)	3 + diseases n (%)		P value
**ACE**			116.07	<.001
0	71 (13.7)	1 (0.4)	4df	
1	31 (6.0)	13 (4.6)		
2	53 (10.2)	12 (4.3)		
3	105 (20.3)	12 (4.3)		
4+	256 (49.8)	244 (86.5)		
**Age**				
60-64	184 (40.7)	78 (27.7)	47.56	<.001
65-69	178 (39.4)	89 (31.6)	4df	
70-74	62 (13.7)	61 (21.6)		
75-79	14 (3.1)	15 (5.3)		
80+	14 (3.1)	39 (13.8)		
**Gender**			4.18	.041
Male	198 (43.8)	102 (36.2)	1df	
Female	254 (56.2)	180 (63.8)		
**Marital status**			29.82	<.001
Never married	5 (1.1)	6 (2.1)	4df	
Currently married	330 (73.0)	153 (54.3)		
Divorced	13 (2.9)	8 (2.8)		
Separated	11 (2.4)	8 (2.8)		
Widowed	93 (20.6)	107 (37.9)		
**Occupation**			79.93	<.001
Government staff	30 (6.6)	6 (2.1)	6df	
Own business	191 (42.3)	90 (31.9)		
Involve in the family business	24 (5.3)	12 (4.3)		
Company staff/worker	26 (5.8)	4 (1.4)		
Dependent	81 (17.9)	133 (47.2)		
Retired	92 (20.4)	36 (12.8)		
Others	8 (1.8)	1 (0.4)		
**Education**			36.56	<.001
Illiterate	248 (54.9)	214 (75.9)	5df	
Can read and write	25 (5.5)	10 (3.5)		
Primary school level	54 (11.9)	20 (7.1)		
Secondary school level	45 (10.0)	19 (6.7)		
Tertiary school	65 (14.4)	18 (6.4)		
Post-graduate	15 (3.3)	1 (0.4)		
**Income**			18.92	<.001
0-15K	269 (59.5)	208 (73.8)	4df	
16-30k	86 (19)	38 (13.5)		
31-45k	18 (4.0)	12 (4.3)		
46-60k	22 (4.9)	5 (1.8)		
>60k	57 (12.6)	19 (6.7)		
**Family structure**			35.80	<.001
Nuclear family	108 (23.9)	33 (11.7)	2df	
Three generation family	65 (14.4)	86 (30.5)		
Extended family	279 (61.7)	163 (57.8)		
**Ethnicity**			3.72	.293
Gwarri	115 (25.4)	78 (27.7)	3df	
Hausa	103 (22.8)	71 (25.2)		
Nupe	137 (30.3)	67 (23.8)		
Others	97 (21.5)	66 (23.4)		

### Adverse childhood experiences and multimorbidity

**Fig 1:**
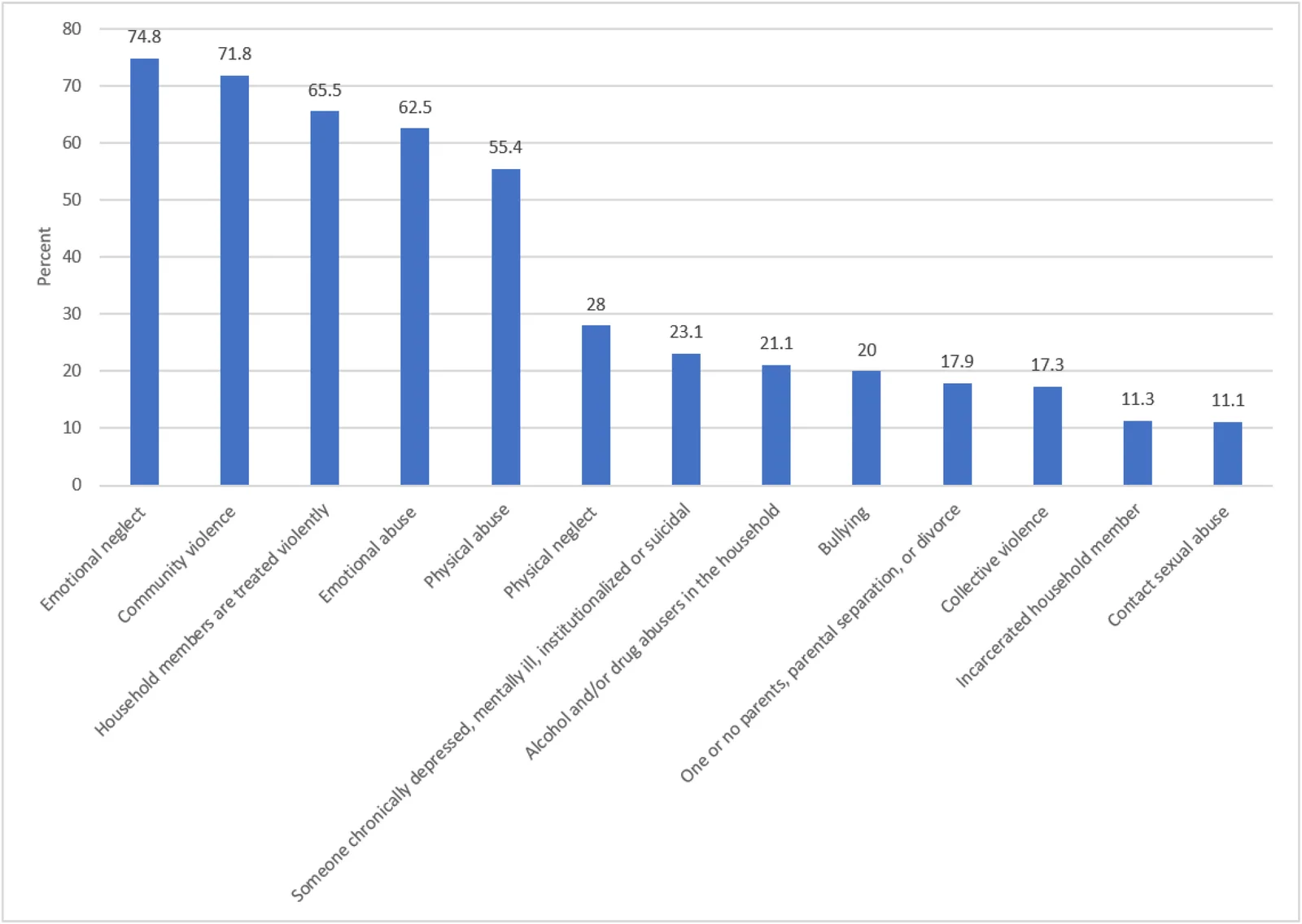
Percent (%) distribution of various types of ACE in the study sample.

The distribution of adverse childhood experiences (ACEs) and multimorbidity is presented in Table 2 and illustrated in Fig 1. A high proportion of participants reported substantial exposure to childhood adversity, with 68.4% experiencing four or more ACEs before the age of 18. Smaller proportions reported three (15.9%), two (8.7%), one (6.0%), or no ACE exposure (0.8%).

Regarding multimorbidity, most participants (61.6%) reported having two chronic conditions, while 31.3% had three conditions. A smaller percentage reported four (5.2%) or five or more (1.9%) chronic conditions.

Overall, both cumulative ACE exposure and the burden of multiple chronic conditions were considerable in this study population (Table 2; Fig 1).

### Bivariate associations with multimorbidity

Bivariate analyses revealed significant associations between multimorbidity and ACE exposure, age, gender, marital status, education, occupation, income, and family structure (p < 0.05). Participants with higher cumulative ACE scores were more likely to report three or more chronic conditions (χ² = 116.07, df = 4, p < 0.001). Advanced age (≥80 years) was associated with higher multimorbidity prevalence (χ² = 47.56, df = 4, p < 0.001). Female participants had slightly higher multimorbidity rates than males (χ² = 4.18, df = 1, p = 0.041). Ethnicity was not significantly associated with multimorbidity (χ² = 3.72, df = 3, p = 0.293) (Table 3).

### Independent predictors of multimorbidity

As shown in Table 4, hierarchical logistic regression demonstrated that cumulative adverse childhood experience (ACE) exposure was a strong independent predictor of multimorbidity. In the unadjusted model (Model 1), participants reporting ≥4 ACEs had substantially higher odds of multimorbidity compared with those with no ACE exposure (OR = 67.14, 95% CI: 9.25–487.04, p = 0.001). After adjustment for socio-demographic variables (Model 2), the association remained statistically significant, although attenuated (OR = 15.58, 95% CI: 1.33–182.21, p = 0.029).

**Table 4:** Multivariable logistic regression analysis of factors associated with multimorbidity.

Variable	Odds ratio	P value	95%CI		Odd ratio	P value	95%CI	
			Lower	Upper			Lower	Upper
**ACE**								
0	1.00				1.00			
1	29.77	.001	3.73	237.67	8.08	.108	.63	103.37
2	16.07	.009	2.02	127.49	3.45	.338	.27	43.42
3	8.11	.047	1.03	63.79	1.66	.693	.13	20.52
4+	67.14	.001	9.25	487.04	15.58	.029	1.33	182.21
**Age**								
60-64					1.00			
65-69					.88	.556	.57	1.34
70-74					1.11	.694	.63	1.95
75-79					1.38	.492	.54	3.48
80+					2.98	.009	1.31	6.75
**Gender**								
Male					1.00			
Female					.93	.726	.62	1.39
**Marital status**								
Never married					1.00			
Currently married					.99	.992	.26	3.74
Divorced					.84	.845	.16	4.43
Separated					.68	.662	.12	3.68
Widowed					.85	.827	.21	3.39
**Occupation**								
Government staff					1.00			
Own business					.93	.921	.26	3.27
Involve in family business					.88	.871	.21	3.67
Company staff/worker					.30	.157	.05	1.59
Dependent					2.82	.122	.75	10.52
Retired					1.52	.474	.47	4.86
Others					.27	.298	.02	3.16
**Education**								
Illiterate					1.00			
Can read and write					.61	.267	.26	1.44
Primary school level					.63	.179	.33	1.22
Secondary school level					.90	.816	.37	2.16
Tertiary school					.30	.050	.09	.99
Post-graduate					.08	.045	.01	.94
**Income**								
0-15K					1.00			
16-30k					.90	.712	.53	1.53
31-45k					2.00	.162	.75	5.30
46-60k					.82	.774	.21	3.12
>60k					2.54	.093	.85	7.58
**Family structure**								
Nuclear family					1.00			
Three generation family					2.15	.015	1.16	4.00
Extended family					1.46	.142	.88	2.42
**Ethnicity**								
Gwarri					1.00			
Hausa					.86	.539	.53	1.39
Nupe					.95	.847	.58	1.54
Others					.76	.299	.46	1.26

Among covariates, advanced age (≥80 years) was independently associated with multimorbidity (OR = 2.98, 95% CI: 1.31–6.75, p = 0.009), as was living in a three-generation household (OR = 2.15, 95% CI: 1.16–4.00, p = 0.015). Higher educational attainment showed an inverse association with multimorbidity. Other factors, including sex, marital status, occupation, income, and ethnicity, were not statistically significant after adjustment. Model diagnostics confirmed adequate goodness-of-fit and no evidence of multicollinearity among covariates.

Overall, the findings indicate that cumulative ACE exposure, alongside advanced age and household structure, are important determinants of multimorbidity in older adults in Nigeria. The magnitude of the ACE effect should be interpreted cautiously given the wide confidence intervals observed, likely reflecting sparse data in some exposure categories and variability in responses, which limits estimate precision.

## Discussion

This study provides novel evidence on the relationship between adverse childhood experiences (ACEs) and multimorbidity among older adults in Niger State, North-Central Nigeria. To our knowledge, it represents one of the first empirical investigations in this setting to examine the long-term implications of cumulative childhood adversity for health in later life. The findings demonstrate a high prevalence of ACE exposure within this cohort and show that cumulative ACEs significantly predict multimorbidity among older adults [18].

The distribution of ACEs observed in this study aligns with global evidence indicating that childhood adversity is widespread across diverse populations. International research consistently demonstrates that ACE exposure cuts across racial and ethnic groups, although the magnitude varies by socioeconomic context [18]. Importantly, earlier studies have shown that exposure to one ACE increases the likelihood of additional adversities, reflecting a clustering pattern that may intensify cumulative risk. This clustering effect strengthens the predictive value of cumulative ACE scores for adverse health outcomes in adulthood [19].

Emotional neglect and exposure to community violence were the most frequently reported ACE domains in this study. Participants with these experiences were more likely to report higher numbers of chronic conditions, suggesting that these specific adversities may be important predictors of later-life multimorbidity [19]. These findings are consistent with previous studies demonstrating that psychosocial stressors during childhood significantly predict chronic disease risk in adulthood [20]. However, variations in the most prevalent ACE domains compared with other Nigerian studies likely reflect regional and sociocultural differences in exposure patterns and reporting practices [21].

The high prevalence of ACEs reported in this cohort must be interpreted cautiously. Although participants were provided sufficient time to recall pre-18 experiences, retrospective reporting may still be influenced by recall bias, particularly among older age groups. Furthermore, because the sample comprised individuals with chronic conditions, the observed distribution of multimorbidity may not fully represent that of the broader population. Educational level may also shape how individuals interpret and report childhood adversity, potentially influencing prevalence estimates [24].

While identifying determinants of ACE exposure was not a primary objective of this study, prior Nigerian research suggests that lower maternal education and lower household wealth significantly **predict** childhood adversity [20]. The predominance of low educational attainment and low income among participants in this study may therefore reflect structural vulnerabilities that increase exposure to ACEs and, in turn, predict poorer health trajectories. These findings suggest a possible intergenerational cycle in which socioeconomic disadvantage increases the likelihood of ACE exposure, which subsequently predicts chronic disease accumulation in later life [20].

The primary objective of this analysis was to evaluate whether childhood adversity predicts multimorbidity among older Nigerian adults. The findings demonstrate a statistically significant moderate positive correlation between cumulative ACE scores and the number of chronic conditions (r = 0.362, p = 0.01). Hierarchical logistic regression further confirmed that cumulative ACE exposure independently predicts multimorbidity. A clear dose–response pattern was observed: individuals reporting four or more ACEs had substantially higher odds of multimorbidity compared with those reporting none, even after adjustment for socio-demographic covariates. Although adjustment reduced the magnitude of the association, cumulative ACE exposure remained a significant independent predictor of multimorbidity [2,22].

These results are consistent with international evidence showing that cumulative ACE exposure predicts multimorbidity, functional decline, and premature mortality in adulthood [2,22]. The observed dose-dependent relationship supports life-course epidemiological models, which posit that early-life adversity exerts long-term biological and behavioral effects that shape disease risk across the lifespan. Chronic stress activation, maladaptive coping behaviors, and socioeconomic disadvantage may act as mediating pathways through which ACEs predict chronic disease clustering in later life [23,24].

Overall, the findings indicate that cumulative childhood adversity significantly predicts multimorbidity among older adults in Nigeria. Integrating ACE screening and trauma-informed approaches into public health policy may enhance early identification of high-risk populations and strengthen chronic disease prevention strategies [2,22]. A life-course approach that prioritizes early intervention may therefore play a critical role in reducing the long-term burden of multimorbidity in low- and middle-income settings [23,24].

### Limitations

This study’s cross-sectional design limits causal inference between adverse childhood experiences (ACEs) and multimorbidity. Retrospective self-reported ACEs are subject to recall bias and may result in both differential and non-differential misclassification. Recall may be influenced by current health status, leading to over-reporting, or by memory decay and social desirability, leading to under-reporting; both could bias effect estimates in either direction. Although chronic conditions were cross-checked where possible using clinical records, some misclassification may persist.

The study was conducted in North-Central Nigeria, which may limit generalizability. In addition, residual confounding from unmeasured factors such as lifestyle and environmental exposures cannot be excluded.

## Conclusion

Cumulative adverse childhood experiences significantly predict multimorbidity among older Nigerian adults. These findings underscore the importance of early-life interventions and life-course approaches. Integrating ACE screening and trauma-informed strategies into healthcare and public health policy may improve early identification of high-risk individuals and inform strategies to prevent chronic disease accumulation.

## Supporting information

S1 Data Quantitative data. (XLS)
