# Supplementary material for: The impact of adverse childhood experiences on the development of multimorbidity in older adults in Nigeria
Source: PLoS One. 2026 Sep 25;21(9):e0358944. doi: 10.1371/journal.pone.0358944 (PMC13614559; doi:10.1371/journal.pone.0358944)
Supplement: Supplementary file 1 [file pone.0358944.s002.pdf]

Comment by Reviewer

1. Conceptual Organization and Coherence

There is a rapid move between topics (multimorbidity burden, polypharmacy, disease trajectories, genetic predispositions, life-course risk accumulation, and ACEs)

-There is full integration of these themes, and as a result, the rationale for linking ACEs to multimorbidity is not developed as clearly as it could be.

Advice: restructure to follow a logical progression from the general burden of multimorbidity to the specific relevance of ACEs.

2. Clarity and Precision of Statements

Some sentences are imprecise or grammatically unclear.

For example, "understanding multimorbidity "has" goes beyond the context of the etiology of a single disease entity" contains errors that hinder readability. - Correct the sentence.

Several phrases such as "this seems the single most important factor" present evaluative claims without citations. - *Provide citation.*

3. Depth and Specificity of Literature Review

The introduction provides multiple references to ACEs literature. – Provide an even synthesis of ACEs literatures.

Important epidemiologic findings are mentioned briefly but not sufficiently put into context. – *work on it.*

For example, the text describes associations between ACEs and outcomes such as hypertension, diabetes, depression, and premature death, but does not link these consistently to multimorbidity or explain hypothesized mechanisms. – *Work on this*

Additionally, statement like “the literature on broader measures of mental health and well-being is less extensive” *need specific citations or clarification*.

4. Justification and Novelty of the Study

The introduction claims that the study “took a step further” than previous research but provides limited detail about how the methodological approach differs from existing work. – *Provide the further steps taken.*

The novelty appears to rest on studying older Nigerian adults with established multimorbidity – *amplify this in the justification.*

Consider these questions to clearly articulate the justification of the research:

1. What gaps in the literature exist specifically in LMIC contexts and among older adults.
2. Why examining ACEs in a multimorbid population is necessary.
3. How the study design addresses previously unanswered questions.

5. Alignment Between Aims and Introduction

The aim, stated as to evaluate whether childhood conditions are associated with “trajectories of chronic conditions,” does not match the cross-sectional nature of the study.

Unless longitudinal data are analyzed, “trajectories” is an inaccurate term. – *Revise* terminology to avoid misleading.

The aims should reflect the actual analytical approach as carried out in the research (Consider “associations between ACE exposure and multimorbidity prevalence among older adults”).

6. Writing Quality and Redundancy

Some paragraphs combine too many distinct ideas, making it difficult to maintain thematic focus. Consider restructuring these paragraphs.

Reviewer Comment on Methods

1. Clarity and Completeness of Study Design

The manuscript refers to the research as "part of a larger study," but does not clearly identify the specific study design. Although some design features can be inferred, the lack of explicit description limits interpretability.

- The authors should clearly state the study design (e.g., cross-sectional, retrospective, observational) up front and describe its relationship to prior published work; summarizing essential methods undermines reproducibility for readers who may not have access to those sources.

Additionally, the sampling strategy is insufficiently detailed. The authors should

- specify how participants were randomly selected from hospital settings,
- state what sampling frame was used.

It is unclear whether the sample represents all older adults with multimorbidity in the region or only those who actively seek care at selected hospitals. - *clarify this, please*

2. Measurement of Variables

The section provides broad descriptions of variables, yet lacks important operational detail.

ACEs measurement: Although the ACE-IQ is named:

- The authors should describe how many items were used, whether the optional modules were included, or whether scoring followed WHO recommendations.

- The phrasing “Questions about ACEs of the respondents’ first 18 years of life” is incomplete.

Multimorbidity measurement:

The authors adopt the NICE definition and use the PURE list of chronic diseases, which is appropriate. However,

- *Authors should clarify whether all diagnoses were physician-confirmed or whether recall was used when records were incomplete.*
- *Authors should justify with methodological references the rationale for counting conditions (simple count method).*

Confounders:

While some socio-demographic variables are listed, the selection rationale is not articulated.

- *Authors should clarify whether lifestyle factors, socioeconomic status, or healthcare access were unavailable or omitted.*
- *Authors should analytically justify why they grouped age into 10-year intervals after entering.*

3. Statistical Analysis and Reporting

The statistical analysis section provides a general overview but lacks the detail required for high-quality reporting.

- *The authors should justify categorizing ACE and multimorbidity scores. Note that categorization of continuous or count variables can reduce statistical power and introduce bias unless supported.*
- *Although the abstract mentions “multiple logistic regression”, this section does not. If regression models were used, details on model specification, covariates, variable*

entry method, model assumptions, multicollinearity checks, and goodness-of-fit should be presented.

- Reference to Cronbach's alpha for "internal consistency of data" is vague. Internal consistency applies to multi-item scales, not entire datasets.

  It is unclear whether Cronbach's alpha was calculated for the ACE-IQ, for socioeconomic scales, or for another instrument. - *This needs precise clarification.*

Additionally, reliance solely on chi-square tests is limited for examining multivariable relationships such as ACE exposure and multimorbidity. – *Clarify*

4. Missing Information Necessary for Reproducibility

Several methodological details essential for reproducibility are absent:

- *Inclusion/exclusion criteria (e.g., cognitive impairment, severe illness).*
- *Procedures for training interviewers, ensuring data quality, and maintaining consistency in diagnosis verification.*
- *Handling of missing data and whether imputation was conducted.*
- *Data collection duration and setting specifics (e.g., outpatient clinics, inpatient units).*
- *Ethical considerations related to sensitive ACE questions in an older adult population, including privacy, distress, and referral pathways.*

These details are required to be able to fully evaluate potential biases or threats to internal validity.

5. Alignment Between Methods and Study Aims

The indicated aim of examining “trajectories of chronic conditions” is inconsistent with the cross-sectional nature of the data, as no longitudinal measurement of disease progression or incidence is mentioned.

- *The Methods section should be revised to align with the actual analytic capabilities of the dataset.*

Reviewer Comment on Results

The Results section provides extensive descriptive statistics and inferential analyses; however, several issues require attention to improve clarity, accuracy, and scientific rigor. The section contains valuable data but would benefit from clearer organization, better reporting standards, and more careful interpretation of findings.

1. Organization and Presentation of Results

While the section presents demographic characteristics, ACE prevalence, multimorbidity patterns, chi-square associations, and logistic regression findings, the narrative is at times disorganized and difficult to follow.

Key messages are buried within long descriptive passages. The results should be structured using subsections or paragraphs (e.g., “Participant Characteristics,” “Prevalence of ACEs and Multimorbidity,” “Bivariate Associations,” “Multivariable Regression Analysis”) to enhance readability

2. Accuracy and Quality of Descriptive Reporting

The descriptive summary is generally adequate; however, several inconsistencies and unclear statements require attention:

- The statement *“contact sexual abuse is found to best the lowest”* appears to be a typographical error and should be corrected for clarity.
- The prevalence of ACEs (e.g., 64.4% for 4+ ACEs) is high and warrants cautious interpretation, particularly given recall bias in older adults; this should be noted in the discussion but clearly reported here.

The tables themselves are informative but lack consistent formatting - tables to be clean and readable.

3. Interpretation and Reporting of Chi-Square Tests

The chi-square tests indicate statistically significant associations between multimorbidity and nearly all socio-demographic variables, except ethnicity. However, the narrative does not discuss effect sizes (e.g., Cramer’s V), which limits understanding of practical significance. Additionally, chi-square tests do not establish directionality or account for confounding. - This limitation should be acknowledged.

4. Logistic Regression Analysis (Clarity and Appropriateness)

The logistic regression results constitute a central finding, yet several concerns arise:

- Extremely large odds ratios (e.g., OR = 67.14, CI: 9.25–487.04) suggest potential issues such as sparse data bias, small cell sizes, or model instability. - These require discussion or sensitivity analyses, or clarification.
- Model development procedures are insufficiently described in Methods; therefore, interpreting these results is challenging.
- The narrative states that “effect… remain unchanged,” but the OR drops from 67.14 to 15.58 after adjustment. - This is not unchanged and should be clarified

- Several covariates have wide confidence intervals (e.g., postgraduate education, OR = 0.08, CI: 0.01-0.94), suggesting imprecision due to small subgroup sizes. - Clarify.
- The authors should clarify whether multicollinearity was assessed; otherwise, estimates may be distorted.
- It is unclear why multimorbidity was dichotomized at 3+ diseases. This categorization lacks justification and significantly affects model outcomes.

Furthermore, both ACE and multimorbidity variables are categorized despite being count variables; the resulting loss of statistical power and increased risk of misclassification should be acknowledged.

5. Reporting Standards

The results contain instances of interpretative language (e.g., "found to have suffered more" or "indicates that ACEs is strongly associated"), which should be reserved for the Discussion. The Results section should focus strictly on data reporting without causal or evaluative statements. – *work on it.*

Provide the following information:

- Confidence intervals for *all* Ors
- Exact p-values (unless <0.001)
- Clear identification of reference categories

6. Logical Consistency

There is an inconsistency between earlier statements (e.g., "majority of older people… suffer from at least 2 diseases") and the dichotomization into *<3 vs. 3+ diseases*. – *Clarify*

Similarly, earlier emphasis on "trajectories" is unsupported by cross-sectional data, and the Results section appropriately reports associations only - avoid implying causality ("effect," "predicts," "net effect").

Reviewer Comment on Discussion and Conclusion

1. Interpretation of Main Findings

The authors restate the principal finding—an association between higher cumulative ACE exposure and greater multimorbidity burden—and relate this to a dose–response pattern consistent with prior literature. This is a strength and aligns well with the stated objectives.

However:

- The Discussion repeatedly implies or borders on causal language (e.g., "ACEs leading to multimorbidity," "prevention of multimorbidity should begin from childhood") despite the cross-sectional design. - reframed this cautiously as an association, not a proven causal pathway.
- The text introduces a "statistically significant positive moderate correlation ($r = 0.362$, $p = 0.01$)" that was not clearly reported or described in the Results section as presented. – Ensure the analytic path (correlation vs logistic regression) is consistent across Results and Discussion.

Clarifying the exact strength and nature of the association - commenting on effect sizes and confidence intervals, not just p-values.

2. Positioning Within Existing Literature

3. Consideration of Bias, Validity, and Alternative Explanations

Key issues that warrant deeper discussion:

- The study includes older adults already engaged with hospital care and with existing multimorbidity, which limits generalizability to community-dwelling older adults and healthier populations. - *Selection bias*
- Individuals with the most severe ACE exposures and poorest health may not have survived to older age, potentially underestimating the true ACE–multimorbidity association. - *Survivor bias*
- While age, marital status, education, and occupation are mentioned, many relevant confounders (e.g., socioeconomic status over the life course, health behaviors, access to healthcare, early-life nutrition) are not accounted for. - *Residual confounding*
- The assumption that “giving enough time to recall” reduces recall bias is questionable; retrospective reporting of ACEs in older adults is inherently vulnerable to underreporting, reinterpretation, or social desirability bias and should be discussed more critically. - *Measurement limitations*
- multimorbidity burden in later life”).

6. Limitations Section

The Limitations subsection is a useful start but is currently too brief and framed somewhat defensively.

Suggested enhancements:

- Explicitly name key limitations: cross-sectional design, retrospective self-report of ACEs, hospital-based sample, high proportion with low education, potential measurement error in multimorbidity classification.
- Acknowledge that the strong ORs and wide confidence intervals in regression analyses may reflect sparse data or instability in some categories.
- Discuss the lack of childhood malnutrition and other early-life indicators (as the authors mention) as a significant gap.

7. Conclusion and "Take-Home" Message

The core conclusion, that higher ACE exposure is associated with worse health profiles and greater multimorbidity in older adults in Niger State, is appropriate and important. However, the conclusion should:

- Use non-causal language (e.g., "associated with" rather than "leading to").
- Explicitly mention the context (hospital-based, multimorbid, older adults), so readers do not overgeneralize.
- Suggest future research directions, such as longitudinal cohort studies, intervention studies, or exploration of mediating pathways (behaviors, psychosocial stress, neurobiological changes).

8. Declarations (Ethics, Funding, Conflict of Interest)

Minor issues to consider:

- There are typographical errors ("Universty," "ob-served") that should be corrected.

- The informed consent statement could be slightly clarified (e.g., explain why consent was obtained 24 hours prior and how voluntary participation and confidentiality were ensured, though this may also fit better in the Methods). - Revise.
