# Supplementary material for: The impact of adverse childhood experiences on the development of multimorbidity in older adults in Nigeria
Source: PLoS One. 2026 Sep 25;21(9):e0358944. doi: 10.1371/journal.pone.0358944 (PMC13614559; doi:10.1371/journal.pone.0358944)
Supplement: Supplementary file 2 [file pone.0358944.s003.docx]

**The Impact of Adverse Childhood Experiences on the Development of Multimorbidity in Older Adults in Nigeria**

1. Abdulsalam Ahmed (ORCID ID 0000-0003-4814-8250)

Department of Public Health/ Community Health, Ibrahim Badamasi Babangida University Lapai Niger state Nigeria & Ranc Orbit International Ltd/Gte, Minna, Niger state. Email: [yatalaa@gmail.com](mailto:yatalaa@gmail.com)

2. Hafiz T.A. Khan (ORCID: 0000-0002-1817-3730)

College of Nursing, Midwifery, and Healthcare, University of West London

Paragon House, Boston Manor Road, Brentford TW8 9GB, London, United Kingdom

Hafiz.Khan@uwl.ac.uk & Oxford Institute of Population Ageing, University of Oxford, Oxford UK [Hafiz.Khan@uwl.ac.uk](mailto:Hafiz.Khan@uwl.ac.uk)

3. Nestor Asiamah (Corresponding author) (ORCID 0000-0002-1157-6430)

Division of Interdisciplinary Research and Practice, School of Health and Social Care, Colchester CO4 3SQ, Essex, United Kingdom; Email: [n.asiamah@essex.ac.uk](mailto:n.asiamah@essex.ac.uk)

**Abstract**

Multimorbidity is becoming a big challenge in the 21^st^ century primarily due to an ageing population across the world. Understanding the determinants of multimorbidity beyond the etiology of a single disease entity is needed. One good starting point is to study multimorbidity using a chronological approach. For example, to uncover the association of Adverse Childhood Experiences (ACEs) and multimorbidity. Thus, this study is aimed to evaluate if childhood conditions are associated with trajectories of chronic conditions among geriatrics population in Niger state north-central Nigeria. Face-to-face data was collected from a cross-section of 734 patients aged 60 years and over with 2 or more chronic diseases who visit for routine outpatient care and consented to participate in the study. The participants were randomly selected from 4 high volume hospitals in Niger state over 5 months (October 2021 to February 2022). The data was inputted electronically via JISC online data collecting tool and exported to IBM SPSS version 27 for analysis. An exploratory data analysis (EDA) was carried out in addition to multiple logistic regression modelling. It has been found that multimorbidity in later life is associated with ACE. Adjusting for age, marital status, educational level, and occupation factors only marginally declined this association. Therefore, the prevention of multimorbidity should begin from childhood by preventing ACEs through the creation of a harmless and positive setting for both children and families and a system that supports healthy families. This is important for policymakers for formulating plans and policy implementation.

***Keywords:*** *Multimorbidity, Adverse Childhood Experiences, Chronic medical conditions, Niger state, Nigeria, Older Adults*

**Introduction**

Multimorbidity is a growing issue and poses a major challenge around the world to population health mainly because it’s linked to complex health needs(1). Likewise, their management portrays a potential complication in waiting because treating all existing diseases could result in a substantial burden of care and polypharmacy if guidelines for treating each individual condition are followed(2). Even though the importance of multimorbidity has long been recognized in clinical care, it has received much less attention than the epidemiology of single-system diseases. Despite the focus on the single disease entity, it is good to realize that understanding multimorbidity has goes beyond the context of the etiology of a single disease entity. What’s more, is that the cumulative effect of diseases might be more substantial than the effect of any one disease(3). One way to shift the paradigm from the focus on a single disease to multimorbidity is to understand the dynamics of the paths that lead to the accumulation of diseases and multimorbidity in an individual. To this end, evolving studies with notable findings have been on the course. For instance, it was stated that the accumulation of health problems that leads to multimorbidity is a complex condition and can occur as a result of a genetic predisposition, or environmental or unknown factors(4). That said, from public health view, the identification of risk factors is an important aspect of the search for prevention and intervention(3). And this seems the single most important factor in the management of multimorbidity. Another key fact to remember is that non-communicable disease risk factors accumulate over the life course, and integrated prevention strategies can and should be used to intervene at all ages and stages of disease progression through both primary care and secondary care(5). One area that is often ignored especially in developing countries like Nigeria and is of utmost importance in the search for the prevention of multimorbidity is ACEs.

ACEs refer to a wide range of circumstances or events that pose a serious threat to a child’s physical or psychological well-being(6). Adverse events in childhood are of great public health concern given the evidence of their long-term impact on health(6). The ACEs are unexpectedly common in the earliest years, they are mostly anonymous and can be identified during childhood by history from children and caretakers(7). ACEs include child maltreatment (e.g., physical, sexual, and verbal abuse) and broader experiences of household dysfunction, such as witnessing violence in the home, parental separation, and growing up in a household affected by substance misuse, mental illness, or criminal behavior. ACEs have not only been associated with increased health risks later in life, but it can also start to manifest their damage as ill-health and somatization during childhood itself(8).

Previous research has also documented the potential of early life conditions to affect the development of chronic conditions later in life, including hypertension, diabetes, and heart disease(9). Another key fact to remember is that individuals that reported an ACE are at increased odds of high-risk behavior such as alcohol consumption, tobacco smoking, risky sexual behavior, and subsequent adverse health outcome like diabetes, stroke, depression, premature death, etc. compared to individuals who have never reported an ACE(10). Moreover, exposure to one ACE can increase the odds of exposure to additional ACEs, indicating a relationship among other ACE exposures(11).

Although the ACEs framework has provided a mechanism for retrospectively measuring childhood adversities and identifying their impact on health in later life. The literature on the impact of ACEs on broader measures of mental health and well-being is less extensive. In other words, studies have not been focusing on pliable risk factors that may have occurred earlier in the life course. And importantly Early life is arguably the most appropriate life phase for preventative efforts(12). It has been well demonstrated that the history of ACEs has a profound impact on physical, emotional, and cognitive development in children and on physical and psychological health in adulthood(13). An equally significant aspect of the evidence from epidemiological and neurobiological studies suggests adverse childhood experiences (ACE) such as sexual and physical abuse and related adverse experiences to be closely related to enduring brain dysfunctions that in turn, affect physical and mental health throughout the lifespan(14). In another proposed pathway, a history of ACEs may lead to disruptions in the neurobiological structure and functions that may persist into adulthood(15).

The proportion of older adults and multimorbidity is on the rise in Nigeria and other parts of the world, it is also true that in most places, inadequate healthcare force does not receive adequate training or possess the necessary skills to deliver appropriate support and care to elderly people(16). Therefore, there is the need for the identification of early life modifiable risk factors like ACEs to help inform early prevention strategies during critical periods of lifespan. This may prevent delay or compress the development and progression of multimorbidity. The compelling argument is that this study took a step further from the previous studies of predicting multimorbidity from ACEs by comparing linear relationships between ACEs and multimorbidity among older adults with a predetermined diagnosis of multimorbidity. This could serve as a good starting point to establish the association of ACEs and multimorbidity. To this end, this study is aimed to evaluate if childhood conditions are associated with trajectories of chronic conditions among older adults. This study contributes to the existing literature on the health effects of ACEs by conducting research among multimorbid older adults in north-central Nigeria.

**Methods**

### This research is part of a larger study, and the detail of the methods are documented in our earlier publications(17,18).

**Ethics approval** Ethical approval was obtained from the College of Nursing, Midwifery, and Healthcare, Research Ethics Panel, Universty of West London (Ethical Approval No. 1055), and authorization to collect data was obtained from the Research, Ethics, and Publication Committee (REPC) of the Hospitals Management Board, Minna, Niger State of Nigeria. All the participants provided written informed consent before participating in the study.

### **Measurement of variables**

The questions on socio-demographic characteristics of participants were collected, which included age, sex, ethnicity, marital status, types of family or composition of the family, level of education, types of occupation, and monthly family income.

Adverse childhood experiences (ACEs) exposure was assessed using the Adverse Childhood Experiences International Questionnaire (ACE-IQ). The ACE questionnaire is a reliable and valid measure of childhood adversity that has been used extensively in large-scale ACE studies. Questions about ACEs of the respondents’ first 18 years of life.

Multimorbidity was operationalized according to the definition recommended by the National Institute for Health and Care Excellence (NICE): the presence of “two or more long-term health conditions. The morbidity was assessed by adopting the list of chronic diseases used in prospective urban and rural epidemiology (PURE) studies because the disease on the list fulfills WHO criteria for chronic diseases. A simple count of individual chronic conditions was used as the approach to measuring multimorbidity, which is also the most common across literature(19). Multimorbidity comprised self-reported conditions diagnosed and confirmation of diagnoses by the patient’s record.

### **Independent variable**

Adverse childhood experiences were used as the predictor variable in this study. 13 ACEs (1. Physical abuse, 2. Emotional, abuse, 3. Contact sexual abuse, 4. Alcohol and/or drug abuser in the household, 5. Incarcerated houses hold members, 6. Living with someone chronically depressed, mentally ill, institutionalized, or suicidal, 7. Household members were treated violently, 8. One or no parent separation or divorce, 9. Emotional neglect, 10. Physical neglect, 11. Bullying, 12. Community violence, and 13. Collective violence) in the respondents’ first 18 years of life were assessed in the study. Responses to each item on the 13 Adverse Childhood Experiences (ACE) were dichotomized yes =1 or no =0 for having the ACE or not respectively.

### **Outcome variable**

Multiple chronic disease condition (multimorbidity) was assessed in the study by assessing 24 chronic conditions, including hypertension, diabetes, peptic ulcer, arthritis, heart failure, stroke, other heart disease, angina, COPD, CLD, obesity, depression, asthma, cataract, chronic renal failure, osteoporosis, glaucoma, tuberculosis, emotional& mental illness, cancer, Alzheimer’s disease, and dementia. Participants were asked, “Have you ever had the disease?”. If they answered “No” or “I don't know”, they were classified as a disease-free group. To be double sure of the diagnosis the respondent’s treatment case note was crosschecked.

### **Confounding factors**

The following variables were recorded and analyzed: age, marital status, educational level, and occupation. Age was entered as a continuous variable but was then grouped into an interval of 10years. Participants responded to marital status as either never married, currently married, divorced, separated, or widow/er. Education level was evaluated for individuals and was categorized as one of the following: Illiterate, can read and write, primary school level, secondary school, Tertiary school, and post-graduate. The occupation was evaluated as to whether the participants are a government staff, own a business, were involved in a family business, are dependent, or retired.

### **Statistical analysis**

Reported data were collated, checked, coded, and entered JISC online data collection tool and were exported to IBM SPSS version 27 for analysis. The data were then cleaned and analyzed using descriptive and inferential statistics. A descriptive and comparative statistical data analysis was processed to answer the research question. Descriptive statistics were used to summarize the overall characteristics of the participants including gender, age, marital status, family structure, educational level, ethnicity, occupation, and level of income. Variables were measured consistently in line with the existing literature for data analysis. The reliability or internal consistency of data was checked with Cronbach alpha before finalizing the data for analysis.

Responses to each item on the 13 Adverse Childhood Experiences (ACE) were dichotomized (yes =1 or no =0) this was then summed to generate a cumulative ACE score for each participant, ranging from 0 to 13. The summed participant’s ACE score was further categorized into 5 groups based on the cumulative ACE scores: 0, 1, 2, 3, and 4 or higher. Multimorbidity was also transformed into categories based on the number of chronic diseases (2, 3, 4, and 5).

### **Test of assumptions**

A [Chi-Square test of independence](https://www.statology.org/chi-square-test-of-independence/) was used to determine a statistically significant difference association between the two categorical variables ACE and Multimorbidity. The assumption required for Chi-square was met because both variables were categorical and every observation in the dataset was independent i.e., the value of one observation in the dataset does not affect the value of any other observation. Similarly, individuals only belong to one cell in the contingency table. That is, cells in the table were mutually exclusive and finally, the expected value of cells in the contingency table was 5 or greater in at least 80% of cells and no cell had an expected value less than 1. It has been found that there is an association between adverse childhood experiences and multimorbidity.

### **Results**

### **Basic information of the participants**

The sociodemographic features of the participants are presented in Table 1. Of the 800 participants contacted for the studies, 734 consented and answered the questionnaire (response rate 91.8%). Of the participants, more than half (About 59.1%) of the respondents were female and the mean age of the sample was 67.3 years. 65.8% were currently married, 62.9% did not have any form of education, 38.1% had their own business, and earning less than fifteen thousand naira (less than 36 dollars at the official rate of 414. 52 as at 27/06/2022) were reported in nearly 65% of the cases (27/06/2022).

| **Table 1: Socio-demographic characteristics of the respondent (n = 734)** | | |
| --- | --- | --- |
| **Variables** | **n** | **%** |
| Gender  Male  Female | 300  434 | 40.9  59.1 |
| Age  60-64  65-69  70-74  75-79  80 and greater | 262  267  123  29  53 | 35.7  36.4  16.8  4.0  7.2 |
| Marital status  Never married  Currently married  Divorced  Separated  Widow/er | 11  483  21  19  200 | 1.5  65.8  2.9  2.6  27.2 |
| Family structure  Nuclear Family  Three Generation Family  Extended Family | 140  150  442 | 19.1  20.5  60.4 |
| Education level  Illiterate  Can read and write  Primary school level  secondary school  Tertiary school  Post-graduate | 462  35  74  64  83  16 | 62.9  4.8  10.1  8.7  11.3  2.2 |
| Occupation  Government staff  Own business  Involve in the family business  Company staff/ worker  Dependent  Retired  Others (specify) | 36  280  36  30  214  128  10 | 4.9  38.1  4.9  4.1  29.2  17.4  1.4 |
| Ethnicity  Gwarri  Hausa  Nupe  Others | 193  174  204  163 | 26.3  23.7  27.8  22.2 |
| Level of income (Naira)  0-15k  16k-30k  31k-45k  46k-60k  greater than 60 | 477  124  30  27  76 | 65.0  16.9  4.1  3.7  10.4 |

Figure 1 shows various types of ACEs among older adults in the study. It has been found that emotional neglect is common (74.8%) followed by community violence which is 71.8%, and Household members are treated violently (65.5%). On the other hand, contact sexual abuse is found to best the lowest, nearly 11.1%.

Table 2 shows the percentage contributions of ACEs and multimorbidity. About 64.4% older adults are found to have suffered from 4 or more ACEs which indicates that incidence of ACEs is common among older adults in Nigeria. On the other hand, majority of older people (61.6%) are found to suffer from at least 2 diseases. The percentage of older adults is declining with higher orders of diseases. Although multimorbidity refers to the coexistence of two or more chronic or long-term conditions to an individual, however for further analysis it is grouped into two categories (less than 3 diseases, 3 or more diseases). The association between selected variables and multimorbidity is displayed in Table 3.

Figure 1: Percent distribution of various type of ACE in the sudy sample.

|  | Percent |
| --- | --- |
| Emotional neglect | 74.8 |
| Community violence | 71.8 |
| Household members are treated violently | 65.5 |
| Emotional abuse | 62.5 |
| Physical abuse | 55.4 |
| Physical neglect | 28 |
| Someone chronically depressed, mentally ill, institutionalized or suicidal | 23.1 |
| Alcohol and/or drug abusers in the household | 21.1 |
| Bullying | 20 |
| One or no parents, parental separation, or divorce | 17.9 |
| Collective violence | 17.3 |
| Incarcerated household member | 11.3 |
| Contact sexual abuse | 11.1 |

**Table 2: ACE and multimorbidity and their percentages.**

| **ACE/Multimorbidity** | **Cases (n)** | **%** |
| --- | --- | --- |
| **ACE** |  |  |
| 0 | 6 | 0.8 |
| 1 | 44 | 6.0 |
| 2 | 65 | 8.7 |
| 3 | 117 | 15.9 |
| 4+ | 502 | 68.4 |
|  |  |  |
| **Multimorbidity** |  |  |
| 2 | 452 | 61.6 |
| 3 | 230 | 31.3 |
| 4 | 38 | 5.2 |
| 5+ | 14 | 1.9 |

It has been found in Table 3 that ACEs is strongly associated with multimorbidity as indicated by Chi-squared test ($\chi^{2}$=116.07, p<.001). which means that multimorbidity in old age is associated with ACEs. Older adults who had experienced 4 or more ACEs are found to have suffered more with 3+ diseases compared to older people with <3 diseases. Other selected covariates such as age, gender, marital status, occupation, education, income, family structure are also found to be statistically associated with multimorbidity. However, ethnicity is found to be not statistically associated with multimorbidity in the study.

**Table 3: Test of associations between multimorbidity and all other selected variables for analysis.**

| Variables | <3 diseases  n (%) | 3+ diseases  n (%) | $\chi^{2} test$ | P value |
| --- | --- | --- | --- | --- |
| **ACE**  0  1  2  3  4+ | 71 (13.7)  31 (6.0)  53 (10.2)  105 (20.3)  256 (49.8) | 1 (0.4)  13 (4.6)  12 (4.3)  12 (4.3)  244 (86.5) | 116.07  4df | <.001 |
| **Age**  60-64  65-69  70-74  75-79  80+ | 184 (40.7)  178 (39.4)  62 (13.7)  14 (3.1)  14 (3.1) | 78 (27.7)  89 (31.6)  61 (21.6)  15 (5.3)  39 (13.8) | 47.56  4df | <.001 |
| **Gender**  Male  Female | 198 (43.8)  254 (56.2) | 102 (36.2)  180 (63.8) | 4.18  1df | .041 |
| **Marital status**  Never married  Currently married  Divorced  Separated  Widowed | 5 (1.1)  330 (73.0)  13 (2.9)  11 (2.4)  93 (20.6) | 6 (2.1)  153 (54.3)  8 (2.8)  8 (2.8)  107 (37.9) | 29.82  4df | <.001 |
| **Occupation**  Government staff  Own business  Involve in the family business  Company staff/worker  Dependent  Retired  Others | 30 (6.6)  191 (42.3)  24 (5.3)  26 (5.8)  81 (17.9)  92 (20.4)  8 (1.8) | 6 (2.1)  90 (31.9)  12 (4.3)  4 (1.4)  133 (47.2)  36 (12.8)  1 (0.4) | 79.93  6df | <.001 |
| **Education**  Illiterate  Can read and write  Primary school level  Secondary school level  Tertiary school  Post-graduate | 248 (54.9)  25 (5.5)  54 (11.9)  45 (10.0)  65 (14.4)  15 (3.3) | 214 (75.9)  10 (3.5)  20 (7.1)  19 (6.7)  18 (6.4)  1 (0.4) | 36.56  5df | <.001 |
| **Income**  0-15K  16-30k  31-45k  46-60k  >60k | 269 (59.5)  86 (19)  18 (4.0)  22 (4.9)  57 (12.6) | 208 (73.8)  38 (13.5)  12 (4.3)  5 (1.8)  19 (6.7) | 18.92  4df | <.001 |
| **Family structure**  Nuclear family  Three generation family  Extended family | 108 (23.9)  65 (14.4)  279 (61.7) | 33 (11.7)  86 (30.5)  163 (57.8) | 35.80  2df | <.001 |
| **Ethnicity**  Gwarri  Hausa  Nupe  Others | 115 (25.4)  103 (22.8)  137 (30.3)  97 (21.5) | 78 (27.7)  71 (25.2)  67 (23.8)  66 (23.4) | 3.72  3df | .293 |

Table 5 shows the results of hierarchical multiple logistic regression analysis. Regression analysis was carried out by considering only ACEs to examine its association with multimorbidity as a dummy dependent variable (1 for 3+ diseases, 0 for <3 diseases). This is considered as Model 1 to identify net effects of ACEs on multimorbidity. The analysis indicates that there is a strong association between ACEs (ACEs score) and multimorbidity. Increases in cumulative ACEs scores were associated with increased prevalence of multimorbidity in the overall sample. In essence, the higher the number of individual adverse childhood experiences, the higher the likelihood of suffering from 3+ diseases. For example, in Model 1, the effect of 4+ ACEs is found to be statistically significant (OR=67.14, 95%CI: 9.25-487.04).

We then ran another regression (Model 2) considering all remaining selected covariates. This allows us to adjust effects of all remaining variables such as age, gender, marital status, occupation, education, income, family structure, and ethnicity. It has been identified that age (80+ years), education (Post graduate level), family structure (three generation family) are found to be statistically associated with 3+ diseases. While adjustments made for selected covariates, effect of 4+ ACEs remain unchanged and it is found to be statistically significant with 3+ diseases (OR=15.58, 95%CI: 1.33-182.21). This indicates that having suffered from 4+ ACEs is associated with multimorbidity among older adults in Nigeria.

**Table 5: Association between multimorbidity and other selected variables.**

| Variable | Odds ratio | P value | 95%CI | |  | | Odd ratio | P value | 95%CI | |
| --- | --- | --- | --- | --- | --- | --- | --- | --- | --- | --- |
|  |  |  | Lower Upper | |  | |  |  | Lower Upper | |
| **ACE**  0  1  2  3  4+ | 1.00  29.77  16.07  8.11  67.14 | .001  .009  .047  .001 | 3.73  2.02  1.03  9.25 | 237.67  127.49  63.79  487.04 | | 1.00  8.08  3.45  1.66  15.58 | | .108  .338  .693  .029 | .63  .27  .13  1.33 | 103.37  43.42  20.52  182.21 |
| **Age**  60-64  65-69  70-74  75-79  80+ |  |  |  |  | | 1.00  .88  1.11  1.38  2.98 | | .556  .694  .492  .009 | .57  .63  .54  1.31 | 1.34  1.95  3.48  6.75 |
| **Gender**  Male  Female |  |  |  |  | | 1.00  .93 | | .726 | .62 | 1.39 |
| **Marital status**  Never married  Currently married  Divorced  Separated  Widowed |  |  |  |  | | 1.00  .99  .84  .68  .85 | | .992  .845  .662  .827 | .26  .16  .12  .21 | 3.74  4.43  3.68  3.39 |
| **Occupation**  Government staff  Own business  Involve in family business  Company staff/worker  Dependent  Retired  Others |  |  |  |  | | 1.00  .93  .88  .30  2.82  1.52  .27 | | .921  .871  .157  .122  .474  .298 | .26  .21  .05  .75  .47  .02 | 3.27  3.67  1.59  10.52  4.86  3.16 |
| **Education**  Illiterate  Can read and write  Primary school level  Secondary school level  Tertiary school  Post-graduate |  |  |  |  | | 1.00  .61  .63  .90  .30  .08 | | .267  .179  .816  .050  .045 | .26  .33  .37  .09  .01 | 1.44  1.22  2.16  .99  .94 |
| **Income**  0-15K  16-30k  31-45k  46-60k  >60k |  |  |  |  | | 1.00  .90  2.00  .82  2.54 | | .712  .162  .774  .093 | .53  .75  .21  .85 | 1.53  5.30  3.12  7.58 |
| **Family structure**  Nuclear family  Three generation family  Extended family |  |  |  |  | | 1.00  2.15  1.46 | | .015  .142 | 1.16  .88 | 4.00  2.42 |
| **Ethnicity**  Gwarri  Hausa  Nupe  Others |  |  |  |  | | 1.00  .86  .95  .76 | | .539  .847  .299 | .53  .58  .46 | 1.39  1.54  1.26 |

### **Discussion**

To the best of our knowledge, this is the first study that analyses the impact of ACEs on the health of older adults in Niger state north-central Nigeria. And it showed that the prevalence of ACEs is common in this part of the world. This is like findings from that ACEs are common across all race/ethnicity groups, though are somewhat disproportionately lower for White, Non-Hispanic and lowest for Asian children(20). They further stated that black children are disproportionately represented among children with ACEs with over 6 in 10 having ACEs, representing 17.4% of all children in the US with ACEs. Studies have shown that children identify with one ACE, have higher chances of additional ACEs.

The commonest domains of ACE in this study are emotional neglect and community violence. That is, compared with participants without emotional neglect and community violence before the age of 18 years, it was observed that multimorbidity (number of chronic diseases) was higher among those that reported a history of emotional neglect and community violence. The finding in this study is consistent with the results of (21). Conversely, it is only partially congruent with a study conducted in Nigeria (22). While it was similarly stated that most respondents reported they had experienced ACEs, differences were observed in the most prevalent ACEs with psychological neglect, physical neglect psychological abuse, and household substance abuse being the most prevalent in their study.

The prevalence of ACEs reported in this study was high, several factors could interplay between increasing and reducing it. Firstly, the participants were given enough time to recall their experiences before the age of 18 years, this goes a long way to reduce recall bias because the questions are retrospective life experiences, and this bias might have been pronounced among the oldest participants. Secondly, previous studies have shown that ACEs were strongly related to multimorbidity and premature mortality and the high ACEs in these studies are not coincidence because this study comprises only participants with multimorbidity. Interpreting the prevalence of multimorbidity must be done with caution because this might be an overrepresentation of multimorbidity in the general population. However, the reported prevalence of ACEs might also be affected by the literacy level of the participants because there may be differences between age cohorts in their understanding of what qualifies as a difficult childhood and their expectations of how childhood should be.

Although the predictors of experiencing ACEs were not one of the research objectives Salawu and Owoaje, (2020) in their study in Nigeria reported that the predictors of experiencing ACEs were having a mother with primary education or lower education and being in the lowest wealth quintile. This is of important because most participants in this study do not have any form of education and were in the lowest wealth quintile and this is a potentially vicious circle of low education and low wealth quintile leading to ACEs and ACEs leading to multimorbidity.

The main objective of this analysis was to evaluate if childhood conditions are associated with trajectories of chronic conditions among older adults in Nigeria. It found out that there is a statistically significant positive moderate correlation between ACEs (ACEs score) and Multimorbidity (individual number of chronic diseases) (*r* = 0.362, *p* = 0.01). Increases in cumulative ACE scores were associated with increased prevalence of multimorbidity in the overall sample. The higher the number of individual adverse experiences, the higher the number of multimorbidity. Adjusting for age, marital status, educational level, and occupation factors only marginally declined this association. This is like several studies that reported the dose-response relationship between the number of ACEs reported and the increase in the prevalence of multimorbidity in adulthood(8,23). The dose-dependent relationship that was observed in our study also corresponds with previous literature that found an association between the amount of exposure to adverse experiences and risky behaviors and increased likelihood of disease conditions(21).

### **Limitation**

The cross‐sectional design nature of the study cannot establish causation between the predictor variable and the outcome variable. However, since ACEs are childhood occurrence it indicates a temporal relationship between ACE exposure and health outcomes measured as of the survey date. Although the ACE measure used in our study is all-inclusive and consists of 13 different survey items, it does not capture other circumstances such as malnutrition during childhood.

**Conclusion**

This study found an association between ACEs and the rising number of chronic diseases which marginally declined by age, marital status, educational level, and occupation level. All things considered, older adults in Niger state who had experienced higher ACEs had worse health profiles. Therefore, the prevention of multimorbidity should begin from childhood by preventing ACEs through the creation of a harmless and positive setting for both children and families and a system that supports a healthy family.

**Conflict of interest**

The authors declare no conflict of interest with respect to the research, authorship, and/or publication of this article.

**Declarations**

**Ethical approval:**

Ethical approval was obtained from the College of Nursing, Midwifery, and Healthcare, Research Ethics Panel, Universty of West London (Ethical Approval No. 1055), and authorization to collect data was obtained from the Research, Ethics, and Publication Committee (REPC) of the Hospitals Management Board, Minna, Niger State of Nigeria. All the participants provided written informed consent before participating in the study.

**Informed Consent Statement**: Participants freely signed their informed consent about 24 h prior to participating in the study, and the individual’s right to withdraw partially or completely was ob‐served

**Funding:** This research received no external funding.

**References**

1.	Pearson-Stuttard J, Ezzati M, Gregg EW. Multimorbidity—a defining challenge for health systems. Lancet Public Heal. 2019;4(12):e599–600.

2.	Woolford SJ, Aggarwal P, Sheikh CJ, Patel HP. Frailty, multimorbidity and polypharmacy. Medicine (Baltimore). 2021;49(3):166–72.

3.	Fortin M, Haggerty J, Almirall J, Bouhali T, Sasseville M, Lemieux M. Lifestyle factors and multimorbidity: a cross sectional study. BMC Public Health. 2014;14:1–8.

4.	Turabian JL. A Theory of Multimorbidity’. Int Res Med Heal Sci. 2020;3(6):9–16.

5.	Head A, Fleming K, Kypridemos C, Pearson-Stuttard J, O’Flaherty M. Multimorbidity: the case for prevention. J Epidemiol Community Heal. 2021;75(3):242–4.

6.	Boullier M, Blair M. Adverse childhood experiences. Paediatr Child Health (Oxford). 2018;28(3):132–7.

7.	Webster EM. The impact of adverse childhood experiences on health and development in young children. Glob Pediatr Heal. 2022;9:2333794X221078708.

8.	Gruenewald TL, Karlamangla AS, Hu P, Stein-Merkin S, Crandall C, Koretz B, et al. History of socioeconomic disadvantage and allostatic load in later life. Soc Sci Med. 2012;74(1):75–83.

9.	Tan H, Zhou H, Chen J, Ren H, Guo Y, Jiang X. Association of early life adversity with cardiovascular disease and its potential mechanisms: a narrative review. Front Public Heal. 2024;12:1341266.

10.	Campbell JA, Walker RJ, Egede LE. Associations between adverse childhood experiences, high-risk behaviors, and morbidity in adulthood. Am J Prev Med. 2016;50(3):344–52.

11.	Felitti VJ, Anda RF, Nordenberg D, Williamson DF, Spitz AM, Edwards V, et al. Relationship of childhood abuse and household dysfunction to many of the leading causes of death in adults: The Adverse Childhood Experiences (ACE) Study. Am J Prev Med. 1998;14(4):245–58.

12.	Dong M, Anda RF, Felitti VJ, Dube SR, Williamson DF, Thompson TJ, et al. The interrelatedness of multiple forms of childhood abuse, neglect, and household dysfunction. Child Abuse Negl. 2004;28(7):771–84.

13.	Tomasdottir MO, Sigurdsson JA, Petursson H, Kirkengen AL, Krokstad S, McEwen B, et al. Self reported childhood difficulties, adult multimorbidity and allostatic load. A cross-sectional analysis of the Norwegian HUNT study. PLoS One. 2015;10(6):e0130591.

14.	Felitti VJ. Adverse childhood experiences and adult health. Acad Pediatr. 2009;9(3):131–2.

15.	Frodl T, Janowitz D, Schmaal L, Tozzi L, Dobrowolny H, Stein DJ, et al. Childhood adversity impacts on brain subcortical structures relevant to depression. J Psychiatr Res. 2017;86:58–65.

16.	Khan HTA, Ahmed S. Healthcare workforce caring for older adults across the world: an overview. Medicina (B Aires). 2017;75–80.

17.	Ahmed A, Khan HTA, Lawal M. Effective hospital care delivery model for older people in Nigeria with multimorbidity: recommendations for practice. In: Healthcare. MDPI; 2022. p. 1268.

18.	Ahmed A, Khan HTA, Lawal M. Patients’ satisfaction with healthcare services among older people with multimorbidity: Subnational gender perspective. J Popul Ageing. 2024;1–14.

19.	Huntley AL, Johnson R, Purdy S, Valderas JM, Salisbury C. Measures of multimorbidity and morbidity burden for use in primary care and community settings: a systematic review and guide. Ann Fam Med. 2012;10(2):134–41.

20.	Bethell CD, Davis MB, Gombojav N, Stumbo S, Powers K. Issue brief: Adverse childhood experiences among US children. Child Adolesc Heal Meas Initiat Johns Hopkins Bloom Sch Public Heal. 2017;

21.	Vásquez E, Quiñones A, Ramirez S, Udo T. Association between adverse childhood events and multimorbidity in a racial and ethnic diverse sample of middle-aged and older adults. Innov aging. 2019;3(2):igz016.

22.	Salawu MM, Owoaje ET. Prevalence and predictors of adverse childhood experiences among youths in rural communities of Oyo State, South-west, Nigeria. J Community Med Prim Heal Care. 2020;32(2):27–41.

23.	Vannorsdall TD, Munro CA. The link between childhood adversity and late-life mental health: evidence for the influence of early-life experiences or illusory correlations? Int Psychogeriatrics. 2017;29(3):357–8.
