# Supplementary material for: The impact of adverse childhood experiences on the development of multimorbidity in older adults in Nigeria
Source: PLoS One. 2026 Sep 25;21(9):e0358944. doi: 10.1371/journal.pone.0358944 (PMC13614559; doi:10.1371/journal.pone.0358944)
Supplement: Supplementary file 3 [file pone.0358944.s004.docx]

**AUTHORS RESPONSE TO REVIEWERS**

We sincerely thank the Reviewer for the detailed and constructive comments. We have carefully revised the manuscript to improve conceptual clarity, methodological transparency, statistical reporting, and interpretative rigor. All changes have been incorporated into the revised manuscript. Below we respond point-by-point.

**RESPONSE TO REVIEWER 1**

**1. Conceptual Organization and Coherence**

**Comment:** Rapid movement between topics; insufficient integration; unclear rationale linking ACEs to multimorbidity.

**Response:**
We have substantially restructured the Introduction to follow a clearer logical progression:

1. Global burden and complexity of multimorbidity
2. Limitations of single-disease models
3. Life-course epidemiological framework
4. Biological and behavioral mechanisms linking early adversity to chronic disease accumulation
5. Specific relevance of ACEs
6. Evidence gaps in LMICs and older populations
7. Clear study aim

This restructuring strengthens the conceptual bridge between multimorbidity and ACEs and clarifies the rationale for examining cumulative childhood adversity as an upstream determinant of multimorbidity in later life.

**2. Clarity and Precision of Statements**

**Comment:** Imprecise and grammatically unclear statements; unsupported evaluative claims.

**Response:**
All grammatical inconsistencies have been corrected, including the previously unclear sentence regarding understanding multimorbidity beyond single-disease etiology.

Evaluative statements such as “this seems the single most important factor” have either been removed or supported with appropriate citations.

The manuscript has undergone careful language editing to improve readability and scientific precision.

**3. Depth and Specificity of Literature Review**

**Comment:** Insufficient synthesis of ACE literature; mechanisms not clearly linked to multimorbidity; some claims lack citation.

**Response:**
We expanded the literature synthesis to:

- Provide balanced discussion of major ACE meta-analyses.
- Explicitly connect ACE-related outcomes (hypertension, diabetes, depression, premature mortality) to multimorbidity accumulation.
- Describe hypothesized mechanisms including:
  - Chronic HPA-axis activation
  - Immune dysregulation
  - Behavioral risk mediation
  - Socioeconomic disadvantage over the life course

Statements previously lacking citations (e.g., regarding mental health literature gaps) have been clarified or supported with references.

**4. Justification and Novelty of the Study**

**Comment:** Insufficient articulation of novelty and LMIC relevance.

**Response:**
We amplified the justification by explicitly stating:

- Limited evidence from LMIC contexts, particularly sub-Saharan Africa.
- Scarcity of ACE–multimorbidity research among older adults.
- Absence of studies focusing on already multimorbid older Nigerian populations.
- Importance of examining ACE exposure in healthcare-engaged older adults where disease burden is high.

We clarified that this study advances the literature by:

- Focusing on an underrepresented population (older Nigerian adults with multimorbidity).
- Using hierarchical regression to examine adjusted associations.
- Integrating life-course theory within an LMIC context.

**5. Alignment Between Aims and Introduction**

**Comment:** “Trajectories” inappropriate for cross-sectional data.

**Response:**
We have revised all instances of “trajectories” to:

“associations between cumulative ACE exposure and multimorbidity prevalence among older adults.”

Causal and longitudinal terminology has been removed.

**6. Writing Quality and Redundancy**

**Response:**
Long paragraphs have been restructured into thematically focused subsections to improve readability.

**METHODS**

**1. Clarity and Completeness of Study Design**

**Comment:** Study design not explicitly stated; insufficient sampling details.

**Response:**
We now explicitly state:

“This was a hospital-based cross-sectional observational study.”

We clarified:

- Participants were recruited from selected public secondary health facilities in Niger State.
- Sampling frame consisted of outpatient registers of older adults receiving care.
- Systematic random sampling was applied within eligible lists.
- The sample represents healthcare-seeking older adults, not the entire community population.

**2. Measurement of Variables**

**ACE Measurement**

We clarified:

- The 13 core ACE-IQ domains were used.
- WHO scoring recommendations were followed.
- Optional modules were not included.
- Internal consistency (Cronbach’s alpha) was calculated specifically for ACE items (value now specified in Methods).

**Multimorbidity**

We clarified:

- Diagnoses were primarily self-reported but cross-verified against medical records when available.
- The simple disease count method was used, consistent with multimorbidity epidemiologic literature (Marengoni et al.).

**Confounders**

We justified inclusion of age, sex, education, occupation, income, family structure, and ethnicity based on prior multimorbidity literature.

We clarified that lifestyle behaviors and life-course SES data were unavailable and are acknowledged as limitations.

Age grouping was used for interpretability and consistency with geriatric epidemiology conventions.

**3. Statistical Analysis**

We clarified:

- Justification for categorizing ACEs (0,1,2,3,4+) based on established dose–response literature.
- Multimorbidity dichotomization (≥3 vs <3) was used to distinguish higher disease burden; this is now justified.
- Logistic regression model specification is now fully described.
- Multicollinearity was assessed using VIF (<5 acceptable).
- Hosmer–Lemeshow test results are now reported.
- Exact p-values are reported unless <0.001.

Cronbach’s alpha is now clearly specified as applying only to ACE-IQ.

**4. Missing Information for Reproducibility**

We added:

- Inclusion criteria: ≥60 years, confirmed ≥2 chronic conditions, cognitively able to consent.
- Exclusion criteria: severe cognitive impairment or critical illness.
- Interviewer training procedures.
- Data collection duration.
- Handling of missing data (complete-case analysis; missing <5%).
- Ethical handling of sensitive ACE questions and referral pathway if distress occurred.

**RESULTS**

**1. Organization**

Results are now structured under:

- Participant Characteristics
- Prevalence of ACEs and Multimorbidity
- Bivariate Associations
- Multivariable Logistic Regression

**2. Descriptive Accuracy**

Typographical errors have been corrected.
Prevalence of ≥4 ACEs is reported consistently (68.4%).

**3. Chi-Square Reporting**

We acknowledge that chi-square tests assess association but not direction or causality.

We now acknowledge absence of effect size measures (Cramer’s V) as a limitation.

**4. Logistic Regression Concerns**

We addressed:

- Large ORs likely reflecting sparse reference category (only 0.8% reported 0 ACEs).
- Wide confidence intervals indicating imprecision.
- OR reduction from 67.14 to 15.58 described accurately as attenuation after adjustment.
- Multicollinearity confirmed acceptable.
- Dichotomization rationale clarified.
- All ORs include 95% CI and reference categories clearly stated.

Language implying “unchanged effect” has been corrected.

**5. Reporting Standards**

Interpretative language has been removed from Results and reserved for Discussion.

Exact p-values are reported.

**6. Logical Consistency**

We clarified that:

- Majority had ≥2 conditions (study inclusion criteria).
- Regression distinguishes ≥3 conditions to identify higher burden.

No causal language remains.

**DISCUSSION**

**1. Causal Language**

All causal phrasing has been replaced with:

- “associated with”
- “linked to”
- “correlated with”

We explicitly state that cross-sectional design precludes causal inference.

**2. Correlation Reporting**

The correlation (r = 0.362) is now clearly described in Results before being interpreted in Discussion.

Effect sizes and CI interpretation are discussed, not only p-values.

**3. Bias and Alternative Explanations**

We expanded discussion of:

- Selection bias (hospital-based sample)
- Survivor bias
- Residual confounding
- Recall bias in older adults
- Measurement limitations
- Sparse data and wide CIs

**4. Limitations Section**

Now explicitly lists:

- Cross-sectional design
- Retrospective ACE recall
- Hospital-based sampling
- Low education profile
- Categorization limitations
- Sparse data bias
- Unmeasured early-life factors

**5. Conclusion**

Conclusion now:

- Uses non-causal language.
- Specifies hospital-based older Nigerian adults.
- Recommends longitudinal cohort research.
- Suggests mediation pathway investigation.
- Calls for life-course research in LMIC settings.

**DECLARATIONS**

Typographical errors corrected.
Consent procedures clarified.
Confidentiality and voluntary participation explicitly described.

**SUMMARY**

We are grateful for the reviewer’s thorough evaluation. The manuscript has been substantially strengthened through:

- Conceptual restructuring
- Methodological clarification
- Improved statistical transparency
- Removal of causal language
- Expanded limitations discussion
- Clearer articulation of novelty

We believe the revised manuscript now meets high standards of clarity, rigor, and reproducibility.

Thank you for your constructive guidance.
