# Supplementary material for: The impact of adverse childhood experiences on the development of multimorbidity in older adults in Nigeria
Source: PLoS One. 2026 Sep 25;21(9):e0358944. doi: 10.1371/journal.pone.0358944 (PMC13614559; doi:10.1371/journal.pone.0358944)
Supplement: Supplementary file 5 [file pone.0358944.s006.docx]

**Response to Editor and Reviewers**

Dear Editor and Reviewers,

We sincerely thank you for your detailed and constructive feedback. We have carefully revised the manuscript to address all comments. Below, we provide a point-by-point response outlining the changes made.

**Editor’s Comments**

**1. Clarification of the larger parent study (Page 3 to 5)**

**Response:**
We have expanded the description of the parent study in the Methods section to ensure the manuscript is self-contained. We explicitly describe the study design, setting, sampling procedures, and participant selection criteria.

**2. Abstract revision and clarification of “chronological approach”**

**Response:**
We agree with this observation. The term “chronological approach” has been removed as it inaccurately implied a longitudinal design. The abstract has been fully revised to reflect the correct **analytical cross-sectional design**, emphasizing that ACEs were assessed retrospectively and multimorbidity measured at the time of survey. All causal language has been removed and replaced with associative terms.

**3. Language and readability improvements**

**Response:**
The manuscript has undergone thorough linguistic revision to improve clarity, conciseness, and readability. Long and complex sentences have been restructured throughout.

**Examples revised:**

- Page 2 sentence has been rewritten as
- “*Adverse Childhood Experiences (ACEs) are increasingly recognized as critical early-life determinants of health across the life course. They encompass potentially traumatic events occurring before the age of 18 years that threaten a child’s physical or psychological well-being*”.
- Page 12 sentence has been rewritten as

*“To our knowledge, this study contributes to the limited empirical evidence from this setting on the long-term health implications of cumulative childhood adversity in later life. The findings indicate a high prevalence of ACE exposure in this cohort and show that higher cumulative ACEs are associated with increased odds of multimorbidity among older adults”*

**4. Methods section elaboration**

Response:

We thank the editor for this important observation. In response, we have substantially revised and restructured the Methods section to ensure clarity, completeness, and reproducibility. The section is now fully self-contained and organized into the following components:

Study design, setting, and ethical approval – clearly specifying the analytical cross-sectional design, study setting, and ethical procedures.

Study population and sampling frame – detailing the source population and eligibility criteria.

Sample size determination – describing the formula used, assumptions, and justification for increasing the sample size.

Sampling procedure – outlining the multistage and systematic random sampling approach.

Data collection procedures – describing questionnaire development, pre-testing, interviewer training, and data collection process.

Measures (variables) – newly expanded to provide detailed operational definitions of:

Outcome variable (multimorbidity)

Exposure variable (ACEs), including ACE-IQ domains, scoring approach, and categorization

Covariates, including socio-demographic factors

Reliability assessment, including Cronbach’s alpha for ACE-IQ

Data analysis – newly elaborated to describe statistical procedures, including:

Descriptive and bivariate analyses

Hierarchical logistic regression model specification

Estimation of odds ratios and 95% confidence intervals

Assessment of multicollinearity and model fit

Handling of missing data (clarified in the revised manuscript)

These revisions directly address the editors concerns by providing sufficient methodological detail to allow replication and improve transparency.

**Reviewer 1**

**1. Use of causal language**

**Response:**
All causal terms (e.g., “predicts,” “leads to,” “demonstrates”) have been replaced with appropriate associative language such as “is associated with” or “is linked to.”

**2. Insufficient methodological detail**

**Response:**
Addressed as noted above. The Methods section has been rewritten to ensure full transparency and reproducibility.

**3. Logistic regression concerns (large ORs, wide CIs, instability)**

**Response:**
We appreciate this important observation. We have now added a detailed explanation in the Discussion:

**Response to Reviewer:**

We thank the reviewer for this important observation regarding the large odds ratios. We have added an explanation in the section. The high odds ratios and wide confidence intervals are likely due to sparse data in some exposure–outcome categories, small subgroup sizes within ACE and multimorbidity strata, and the categorization of variables, which can inflate effect estimates. In addition, the hospital-based sample may have introduced selection effects toward individuals with higher disease burden. We have clarified that the magnitude of the odds ratios should be interpreted with caution, and that greater emphasis should be placed on the direction and consistency of the association rather than the size of the estimates.

**4. Justification for ≥3 disease cutoff**

**Response:**
We have now justified this threshold by:

- Referencing prior multimorbidity literature where ≥3 conditions indicate higher clinical complexity
- Explaining that the cutoff reflects greater healthcare burden and functional decline, and aligns with the distribution of conditions in our dataset

**5. Interpretative language in Results**

**Response:**
All interpretative statements have been removed from the Results section. The section now strictly reports statistical findings without inference.

**6. Overstated novelty claims**

**Response:**
The statement has been revised to a more cautious and appropriate form:
*“This study contributes to the limited body of evidence on the association between ACEs and multimorbidity among older adults in sub-Saharan Africa.”*

**7. Bias and limitations**

**Response:**
The limitations section has been substantially expanded to include:

- **Selection bias** (hospital-based sample)
- **Survivor bias** (older adults who survived to ≥60 years)
- **Recall bias** (retrospective ACE reporting)
- **Residual confounding** (unmeasured variables such as early-life socioeconomic status)

We also emphasize the limitation of causal inference due to the cross-sectional design.

**Reviewer 2**

**1. Large odds ratio concern**

**Response:**
We have addressed this concern in both the Results (clarification) and Discussion sections. Specifically:

- We clarify that the large odds ratios may be influenced by sparse data and categorical grouping
- We emphasize cautious interpretation of effect size
- We avoid overstating the magnitude and focus on direction and consistency of association
