# Supplementary material for: The impact of adverse childhood experiences on the development of multimorbidity in older adults in Nigeria
Source: PLoS One. 2026 Sep 25;21(9):e0358944. doi: 10.1371/journal.pone.0358944 (PMC13614559; doi:10.1371/journal.pone.0358944)
Supplement: Supplementary file 6 [file pone.0358944.s007.docx]

**The Impact of Adverse Childhood Experiences on the Development of Multimorbidity in Older Adults in Nigeria**

1.	**Abdulsalam Ahmed (ORCID ID 0000-0003-4814-8250)**

Department of Public Health/ Community Health, Newgate University Minna, Niger state Nigeria & Ranc Orbit International Ltd/Gte, Minna, Niger state. Email: yatalaa@gmail.com

2.	**Hafiz T.A. Khan (ORCID: 0000-0002-1817-3730)**

College of Nursing, Midwifery, and Healthcare, University of West London

Paragon House, Boston Manor Road, Brentford TW8 9GB, London, United Kingdom

Hafiz.Khan@uwl.ac.uk & Oxford Institute of Population Ageing, University of Oxford, Oxford UK, [hafiz.khan@ageing.ox.ac.uk](mailto:hafiz.khan@ageing.ox.ac.uk)

3.	**Nestor Asiamah (Corresponding author) (ORCID 0000-0002-1157-6430)**

Division of Interdisciplinary Research and Practice, School of Health and Social Care, Colchester CO4 3SQ, Essex, United Kingdom; Email: n.asiamah@essex.ac.uk

**Abstract**

**Background:**

Adverse Childhood Experiences (ACEs) are established life-course determinants of adult health, yet evidence on their association with multimorbidity in sub-Saharan Africa remains limited. Nigeria is undergoing rapid demographic and epidemiological transitions, including population ageing and a rising burden of non-communicable diseases. This study examined the association between cumulative ACE exposure and multimorbidity among older adults in North-Central Nigeria.

**Methods:**

A cross-sectional study was conducted among 734 adults aged ≥60 years in Niger State, Nigeria. ACE exposure before age 18 was assessed using the Adverse Childhood Experiences International Questionnaire (ACE-IQ) across 13 domains. Multimorbidity was defined as the presence of ≥2 chronic conditions (NICE criteria) from 24 conditions and analysed as both a count and a categorical variable (≥3 vs. <3 conditions). Chi-square tests assessed bivariate associations, and hierarchical logistic regression models estimated adjusted odds ratios (ORs) with 95% confidence intervals (CIs).

**Results:**

Overall, 68.4% of participants reported ≥4 ACEs, and 38.4% had ≥3 chronic conditions. ACE exposure was significantly associated with multimorbidity (χ² = 116.07, p < 0.001). In unadjusted analysis, ≥4 ACEs were associated with higher odds of multimorbidity (OR = 67.14; 95% CI: 9.25–487.04). After adjustment for socio-demographic factors, the association remained significant (OR = 15.58; 95% CI: 1.33–182.21). Advanced age (≥80 years) and living in three-generation households were also independently associated with multimorbidity.

**Conclusions:**

Cumulative childhood adversity is significantly associated with multimorbidity among older adults in Nigeria, showing a dose–response pattern. These findings support life-course and trauma-informed approaches to reducing the long-term burden of chronic disease in low- and middle-income settings.

**Keywords:** Adverse Childhood Experiences; Multimorbidity; Older Adults; Life-course Epidemiology; Nigeria; Non-communicable Diseases.

**Background**

Adverse Childhood Experiences (ACEs) are increasingly recognized as critical early-life determinants of health across the life course. They encompass potentially traumatic events occurring before the age of 18 years that threaten a child’s physical or psychological well-being. These include child maltreatment (physical, emotional, or sexual abuse) and household dysfunction such as exposure to domestic violence, parental separation, substance misuse, mental illness, or incarceration within the household(1). Since the landmark ACE Study, evidence has consistently demonstrated a graded relationship between cumulative childhood adversity and adverse health outcomes in adulthood(2). Recent systematic reviews have reinforced this, showing that individuals with multiple ACEs are at significantly higher risk of chronic diseases in later life(3).

A substantial body of epidemiological research shows that individuals exposed to ACEs are at increased risk of chronic diseases, including hypertension, diabetes mellitus, cardiovascular diseases, and depression(2,3). ACE exposure is also associated with higher likelihood of engaging in health-risk behaviors—such as tobacco use, harmful alcohol consumption, physical inactivity, and risky sexual behavior—which further elevate the risk of non-communicable diseases (NCDs) and premature mortality (3,5). Importantly, ACEs frequently co-occur; exposure to one adverse event increases the likelihood of exposure to others, leading to cumulative biological and psychosocial stress(3,4). Neurobiological evidence suggests that early-life toxic stress can result in long-term dysregulation of neuroendocrine, immune, and metabolic systems, thereby increasing vulnerability to chronic disease across the lifespan(5).

These long-term consequences are particularly relevant within the framework of multimorbidity, defined as the coexistence of two or more chronic conditions in an individual(6). Globally, multimorbidity has emerged as a major public health challenge driven by population aging and epidemiological transition(6). It is associated with increased healthcare utilization, reduced quality of life, polypharmacy, functional decline, and higher mortality(7). Clinical management is complex because healthcare systems remain largely structured around single-disease models, and applying disease-specific guidelines to individuals with multiple conditions often results in treatment burden and fragmented care(8).

Understanding multimorbidity requires a life-course perspective that goes beyond single-disease etiology. Evidence suggests that the accumulation of chronic conditions is shaped by genetic predisposition, environmental exposures, behavioral risk factors, and social determinants of health(9). From a public health standpoint, identifying early-life modifiable risk factors is crucial for prevention. Risk factors for NCDs accumulate across the lifespan, and early intervention during critical developmental windows may delay or compress the onset of chronic diseases in later life(10). ACEs represent one such early-life exposure that may contribute to disease accumulation and multimorbidity in adulthood(3,4).

In low- and middle-income countries (LMICs), including Nigeria, the relevance of this life-course approach is particularly pronounced. Nigeria is undergoing demographic and epidemiological transitions characterized by a growing population of older adults and a rising burden of NCDs(11). NCDs account for approximately one-third of adult mortality in Nigeria, with cardiovascular diseases and diabetes among the leading contributors(11). As survival improves and life expectancy increases, the prevalence of multimorbidity is also rising. However, the healthcare system remains largely oriented toward acute and single-disease management, with limited geriatric specialization and insufficient integration of preventive life-course strategies.

Despite evidence that childhood adversity is prevalent in many LMIC contexts, including poverty, exposure to violence, family instability, and limited social protection, there is limited empirical research examining the contribution of ACEs to multimorbidity among older adults in Nigeria. Most existing evidence on ACEs and chronic disease originates from high-income settings, limiting contextual applicability to African populations(3,4).

Previous studies have consistently shown that cumulative exposure to adverse childhood experiences (ACEs) is associated with an increased risk of chronic disease and multimorbidity in later life (2–4). Evidence indicates a dose–response relationship, where individuals with a higher number of ACEs are more likely to develop multiple chronic conditions compared with those with few or no ACEs. These associations persist even after accounting for sociodemographic factors such as age, gender, education, and family structure. Earlier research underscores that ACEs contribute to multimorbidity through both behavioral and biological pathways, highlighting the importance of life-course approaches and early preventive strategies to mitigate long-term health risks(2–4). Identifying ACEs as an upstream determinant of multimorbidity has important implications for policy, prevention, and clinical care. Early interventions targeting children at risk of adversity may reduce the future burden of chronic disease and multimorbidity, particularly in LMICs where healthcare systems face resource constraints(3,4,9).

Biological and neurodevelopmental evidence provides plausible mechanisms linking adverse childhood experiences (ACEs) to later-life health outcomes. Research indicates that early adversity can induce lasting alterations in brain structure and function, with consequences for physical and mental health across the lifespan(12). These neurobiological disruptions may persist into adulthood, increasing vulnerability to chronic disease development and progression(12). Although the ACE framework has been widely used to retrospectively assess childhood adversity and its long-term health effects(13), comparatively less attention has been paid to broader measures of mental health, well-being, and modifiable life-course risk factors. Importantly, early life represents a critical window for preventive intervention(2).

Against the backdrop of a rapidly ageing population and rising multimorbidity in Nigeria and globally, health systems, often constrained by limited geriatric expertise, face increasing challenges (14). Identifying modifiable early-life determinants such as ACEs may therefore inform prevention strategies aimed at delaying or compressing multimorbidity. Extending previous research, this study examined the association between cumulative ACE exposure and multimorbidity, including whether a graded (dose–response) pattern exists between ACE burden and multimorbidity among older adults with established chronic conditions. By doing so, it contributes to the growing evidence base on the long-term health implications of childhood adversity in North-Central Nigeria.

**Methods**

**Study Design and Ethical Approval**

This clinic-based geriatric study was conducted as part of a larger investigation, with detailed methodology reported in previous publications (15–17). It employed a hospital-based analytical cross-sectional design conducted in Niger State, North-Central Nigeria, between October 2021 and February 2022. Niger State is geographically divided into three senatorial districts and comprises both urban and rural populations with varying socioeconomic characteristics.

The study was conducted in four high-volume secondary health facilities located in Minna, Bida, Kontagora, and Suleja. These facilities were purposively selected to ensure representation across all senatorial districts and the state capital. All hospitals are managed by the Niger State Hospital Management Board and serve as major referral centres providing outpatient services to a large population of older adults with chronic conditions.

**Study population and sampling frame**

The study population comprised adults aged ≥60 years attending outpatient departments of the selected hospitals. Based on 2020 hospital records, approximately 105,000 patients aged ≥60 years accessed outpatient services across the four facilities, forming the sampling frame.

Eligible participants were those aged ≥60 years, attending outpatient clinics during the study period, diagnosed with at least two chronic medical conditions, and able to provide informed consent and respond to study procedures.

**Sample size determination**

Sample size was calculated using the standard formula for finite populations greater than 10,000:

$$n=\frac{N}{1+N(e^{2})}$$

where *n* is the required sample size, *N* is the population size (105,000), and *e* is the margin of error (0.05). This yielded an initial sample size of approximately 400 participants. To improve statistical power, reduce sampling error, and account for potential non-response or incomplete responses, the sample size was increased to 800.

**Sampling procedure**

A multistage sampling approach was used. First, four hospitals were purposively selected to ensure geographic representation across the state. Second, within each hospital, eligible participants were selected using systematic random sampling from outpatient attendance registers.

Every *k-th* eligible patient meeting inclusion criteria was selected until the required sample size was achieved proportionally across the four facilities.

**Data collection procedures**

Data were collected using a structured, pre-coded questionnaire administered through face-to-face interviews. The instrument was developed based on existing literature and adapted to the Nigerian context to ensure cultural and contextual relevance.

The questionnaire was pre-tested in the same hospital settings prior to the main study. Feedback from the pilot study was used to refine wording, flow, and clarity. Data collection was conducted by the principal investigator and trained research assistants. All participants provided written informed consent prior to participation and were informed of their right to withdraw at any stage without penalty. Confidentiality and anonymity were strictly maintained. Interviews were conducted in English or Hausa, depending on participant preference, to minimize language barriers and enhance response accuracy. Data were captured using the JISC online data collection platform (University of West London), ensuring secure storage and structured data entry.

Ethical approval was obtained from the College of Nursing, Midwifery, and Healthcare Research Ethics Panel, University of West London (Approval No. 1055), and authorization to collect data was granted by the Research, Ethics, and Publication Committee (REPC) of the Hospitals Management Board, Minna, Niger State, Nigeria. Written informed consent was obtained from all participants prior to partaking.

**Measurement of Variables**

**Socio-Demographic Characteristics**

Participant demographics collected included age, sex, ethnicity, marital status, family structure, education level, occupation, and monthly household income. Age was treated as a continuous variable and later grouped into 10-year intervals. Marital status was classified as never married, currently married, divorced, separated, or widowed. Education level was categorized from illiterate to postgraduate. Occupation was classified as government employee, business owner, family business worker, company staff/worker, dependent, retired, or other. Family structure was grouped as nuclear, three-generation, or extended family.

**Adverse Childhood Experiences (ACEs)**

ACE exposure was measured using the Adverse Childhood Experiences International Questionnaire (ACE-IQ), a validated instrument widely used in large-scale studies of childhood adversity. Participants reported experiences before age 18 across 13 domains: physical abuse, emotional abuse, sexual abuse, household alcohol or drug abuse, incarcerated household member, living with someone chronically depressed, mentally ill, institutionalized, or suicidal, household violence, parental separation/divorce, emotional neglect, physical neglect, bullying, community violence, and collective violence. Responses were dichotomized (yes = 1, no = 0), summed to generate a cumulative ACE score (0–13), and categorized into five groups: 0, 1, 2, 3, and ≥4.

**Multimorbidity**

Multimorbidity was defined according to NICE guidelines as the presence of two or more chronic conditions. A total of 24 conditions were assessed, including hypertension, diabetes, peptic ulcer, arthritis, heart failure, stroke, angina, COPD, chronic liver disease, obesity, depression, asthma, cataract, chronic renal failure, osteoporosis, glaucoma, tuberculosis, cancer, Alzheimer’s disease, dementia, and other mental or emotional illnesses. Self-reported diagnoses were cross-checked against medical records when available. Multimorbidity was analyzed both as a simple count of chronic conditions and categorized as 2, 3, 4, or ≥5 conditions.

**Confounding Factors**

Potential confounders included age, sex, marital status, education, occupation, family structure, ethnicity, and income. These variables were included in multivariable analyses to adjust for their potential influence on multimorbidity.

**Statistical Analysis**

Data were collated, coded, and entered using the JISC online platform, then exported to IBM SPSS version 27. Descriptive statistics summarized participant characteristics, ACE exposure, and multimorbidity status. Categorical variables were expressed as frequencies and percentages.

Bivariate associations between multimorbidity and selected variables were assessed using Chi-square (χ²) tests, with Fisher’s exact test applied where expected counts were <5. Variables examined included age, gender, marital status, family structure, education, occupation, income, ethnicity, and ACE score. Statistical significance was set at p < 0.05.

Hierarchical logistic regression analysis was performed to examine factors associated with multimorbidity (≥3 chronic conditions). Two models were specified: Model 1 included only ACE variables to estimate unadjusted associations, while Model 2 included ACEs and socio-demographic covariates (age, sex, marital status, education, occupation, income, family structure, and ethnicity) to account for potential confounding.

ACE scores were categorized as 0, 1, 2, 3, and ≥4 exposures. Odds ratios (ORs) with 95% confidence intervals (CIs) were reported. Model fit was assessed using the VIF, and multicollinearity was evaluated using variance inflation factors (VIFs), with values <5 considered acceptable.

**Results**

**Participant Characteristics**

A total of 734 older adults participated. The majority were female (59.1%) and aged 60–69 years (72.1%). Most were currently married (65.8%) and lived in extended families (60.4%). Regarding education, 62.9% were illiterate, and only 2.2% had postgraduate qualifications. Common occupations included business ownership (38.1%) and dependence on family support (29.2%). Participants were predominantly of Nupe (27.8%) and Gwarri (26.3%) ethnicity. Household income was generally low, with 65% earning ≤15,000 Naira monthly. High cumulative ACE exposure was reported, with 68.4% experiencing four or more ACEs. Multimorbidity was common, with 38.4% reporting three or more chronic conditions (Tables 1 and 3).

| **Table 1. Socio-demographic characteristics of study participants (N = 734)** | | |
| --- | --- | --- |
| **Variables** | **n** | **%** |
| Gender  Male  Female | 300  434 | 40.9  59.1 |
| Age  60-64  65-69  70-74  75-79  80 and greater | 262  267  123  29  53 | 35.7  36.4  16.8  4.0  7.2 |
| Marital status  Never married  Currently married  Divorced  Separated  Widow/er | 11  483  21  19  200 | 1.5  65.8  2.9  2.6  27.2 |
| Family structure  Nuclear Family  Three Generation Family  Extended Family | 140  150  442 | 19.1  20.5  60.4 |
| Education level  Illiterate  Can read and write  Primary school level  secondary school  Tertiary school  Post-graduate | 462  35  74  64  83  16 | 62.9  4.8  10.1  8.7  11.3  2.2 |
| Occupation  Government staff  Own business  Involve in the family business  Company staff/ worker  Dependent  Retired  Others (specify) | 36  280  36  30  214  128  10 | 4.9  38.1  4.9  4.1  29.2  17.4  1.4 |
| Ethnicity  Gwarri  Hausa  Nupe  Others | 193  174  204  163 | 26.3  23.7  27.8  22.2 |
| Level of income (Naira)  0-15k  16k-30k  31k-45k  46k-60k  greater than 60 | 477  124  30  27  76 | 65.0  16.9  4.1  3.7  10.4 |

**Adverse Childhood Experiences and Multimorbidity**

The distribution of adverse childhood experiences (ACEs) and multimorbidity is presented in **Table 2** and illustrated in **Figure 1**. A high proportion of participants reported substantial exposure to childhood adversity, with 68.4% experiencing four or more ACEs before the age of 18. Smaller proportions reported three (15.9%), two (8.7%), one (6.0%), or no ACE exposure (0.8%).

Regarding multimorbidity, most participants (61.6%) reported having two chronic conditions, while 31.3% had three conditions. A smaller percentage reported four (5.2%) or five or more (1.9%) chronic conditions.

Overall, both cumulative ACE exposure and the burden of multiple chronic conditions were considerable in this study population (Table 2; Figure 1).

**Table 2. Frequency and percentage distribution of ACE exposure and multimorbidity.**

| **ACE/Multimorbidity** | **Cases (n)** | **%** |
| --- | --- | --- |
| **ACE** |  |  |
| 0 | 6 | 0.8 |
| 1 | 44 | 6.0 |
| 2 | 65 | 8.7 |
| 3 | 117 | 15.9 |
| 4+ | 502 | 68.4 |
|  |  |  |
| **Multimorbidity** |  |  |
| 2 | 452 | 61.6 |
| 3 | 230 | 31.3 |
| 4 | 38 | 5.2 |
| 5+ | 14 | 1.9 |

Figure 1: Percent distribution of various type of ACE in the sudy sample.

|  | Percent |
| --- | --- |
| Emotional neglect | 74.8 |
| Community violence | 71.8 |
| Household members are treated violently | 65.5 |
| Emotional abuse | 62.5 |
| Physical abuse | 55.4 |
| Physical neglect | 28 |
| Someone chronically depressed, mentally ill, institutionalized or suicidal | 23.1 |
| Alcohol and/or drug abusers in the household | 21.1 |
| Bullying | 20 |
| One or no parents, parental separation, or divorce | 17.9 |
| Collective violence | 17.3 |
| Incarcerated household member | 11.3 |
| Contact sexual abuse | 11.1 |

**Bivariate Associations with Multimorbidity**

Bivariate analyses revealed significant associations between multimorbidity and ACE exposure, age, gender, marital status, education, occupation, income, and family structure (p < 0.05). Participants with higher cumulative ACE scores were more likely to report three or more chronic conditions (χ² = 116.07, df = 4, p < 0.001). Advanced age (≥80 years) was associated with higher multimorbidity prevalence (χ² = 47.56, df = 4, p < 0.001). Female participants had slightly higher multimorbidity rates than males (χ² = 4.18, df = 1, p = 0.041). Ethnicity was not significantly associated with multimorbidity (χ² = 3.72, df = 3, p = 0.293) (Table 3).

**Table 3. Bivariate associations between multimorbidity and selected independent variables.**

| Variables | <3 diseases  n (%) | 3+ diseases  n (%) | $\chi^{2} test$ | P value |
| --- | --- | --- | --- | --- |
| **ACE**  0  1  2  3  4+ | 71 (13.7)  31 (6.0)  53 (10.2)  105 (20.3)  256 (49.8) | 1 (0.4)  13 (4.6)  12 (4.3)  12 (4.3)  244 (86.5) | 116.07  4df | <.001 |
| **Age**  60-64  65-69  70-74  75-79  80+ | 184 (40.7)  178 (39.4)  62 (13.7)  14 (3.1)  14 (3.1) | 78 (27.7)  89 (31.6)  61 (21.6)  15 (5.3)  39 (13.8) | 47.56  4df | <.001 |
| **Gender**  Male  Female | 198 (43.8)  254 (56.2) | 102 (36.2)  180 (63.8) | 4.18  1df | .041 |
| **Marital status**  Never married  Currently married  Divorced  Separated  Widowed | 5 (1.1)  330 (73.0)  13 (2.9)  11 (2.4)  93 (20.6) | 6 (2.1)  153 (54.3)  8 (2.8)  8 (2.8)  107 (37.9) | 29.82  4df | <.001 |
| **Occupation**  Government staff  Own business  Involve in the family business  Company staff/worker  Dependent  Retired  Others | 30 (6.6)  191 (42.3)  24 (5.3)  26 (5.8)  81 (17.9)  92 (20.4)  8 (1.8) | 6 (2.1)  90 (31.9)  12 (4.3)  4 (1.4)  133 (47.2)  36 (12.8)  1 (0.4) | 79.93  6df | <.001 |
| **Education**  Illiterate  Can read and write  Primary school level  Secondary school level  Tertiary school  Post-graduate | 248 (54.9)  25 (5.5)  54 (11.9)  45 (10.0)  65 (14.4)  15 (3.3) | 214 (75.9)  10 (3.5)  20 (7.1)  19 (6.7)  18 (6.4)  1 (0.4) | 36.56  5df | <.001 |
| **Income**  0-15K  16-30k  31-45k  46-60k  >60k | 269 (59.5)  86 (19)  18 (4.0)  22 (4.9)  57 (12.6) | 208 (73.8)  38 (13.5)  12 (4.3)  5 (1.8)  19 (6.7) | 18.92  4df | <.001 |
| **Family structure**  Nuclear family  Three generation family  Extended family | 108 (23.9)  65 (14.4)  279 (61.7) | 33 (11.7)  86 (30.5)  163 (57.8) | 35.80  2df | <.001 |
| **Ethnicity**  Gwarri  Hausa  Nupe  Others | 115 (25.4)  103 (22.8)  137 (30.3)  97 (21.5) | 78 (27.7)  71 (25.2)  67 (23.8)  66 (23.4) | 3.72  3df | .293 |

**Independent Factors Associated with Multimorbidity** As shown in Table 4, hierarchical logistic regression analysis indicated that cumulative ACE exposure was independently associated with multimorbidity. In the unadjusted model (Model 1), participants reporting four or more ACEs had substantially higher odds of multimorbidity compared with those reporting no ACEs (OR = 67.14, 95% CI: 9.25–487.04, p = 0.001). After adjustment for socio-demographic covariates (Model 2), this association remained statistically significant (OR = 15.58, 95% CI: 1.33–182.21, p = 0.029).

Advanced age (≥80 years) was also independently associated with multimorbidity (OR = 2.98, 95% CI: 1.31–6.75, p = 0.009), and living in a three-generation household was similarly associated with higher odds (OR = 2.15, 95% CI: 1.16–4.00, p = 0.015). Higher education levels were associated with lower odds of multimorbidity. Other socio-demographic factors, including sex, marital status, occupation, income, and ethnicity, were not statistically significant after adjustment. The relatively large odds ratios observed in this study should be interpreted with caution. This may be explained by sparse data in some exposure–outcome categories, small subgroup sizes within ACE and multimorbidity strata, and the categorization of variables, all of which can inflate effect estimates and widen confidence intervals. In addition, the hospital-based nature of the sample may have introduced selection effects, with a higher concentration of individuals experiencing more severe or multiple chronic conditions. Consequently, the magnitude of the odds ratios should be interpreted cautiously, with greater emphasis placed on the direction and consistency of the associations rather than the size of the estimates.

Overall, these results indicate that both cumulative ACE exposure and certain socio-demographic factors, particularly advanced age and family structure, contribute significantly to multimorbidity among older adults in Nigeria.

**Table 4. Multivariable logistic regression analysis of factors associated with multimorbidity.**

| Variable | Odds ratio | P value | 95%CI | |  | | Odd ratio | P value | 95%CI | |
| --- | --- | --- | --- | --- | --- | --- | --- | --- | --- | --- |
|  |  |  | Lower Upper | |  | |  |  | Lower Upper | |
| **ACE**  0  1  2  3  4+ | 1.00  29.77  16.07  8.11  67.14 | .001  .009  .047  .001 | 3.73  2.02  1.03  9.25 | 237.67  127.49  63.79  487.04 | | 1.00  8.08  3.45  1.66  15.58 | | .108  .338  .693  .029 | .63  .27  .13  1.33 | 103.37  43.42  20.52  182.21 |
| **Age**  60-64  65-69  70-74  75-79  80+ |  |  |  |  | | 1.00  .88  1.11  1.38  2.98 | | .556  .694  .492  .009 | .57  .63  .54  1.31 | 1.34  1.95  3.48  6.75 |
| **Gender**  Male  Female |  |  |  |  | | 1.00  .93 | | .726 | .62 | 1.39 |
| **Marital status**  Never married  Currently married  Divorced  Separated  Widowed |  |  |  |  | | 1.00  .99  .84  .68  .85 | | .992  .845  .662  .827 | .26  .16  .12  .21 | 3.74  4.43  3.68  3.39 |
| **Occupation**  Government staff  Own business  Involve in family business  Company staff/worker  Dependent  Retired  Others |  |  |  |  | | 1.00  .93  .88  .30  2.82  1.52  .27 | | .921  .871  .157  .122  .474  .298 | .26  .21  .05  .75  .47  .02 | 3.27  3.67  1.59  10.52  4.86  3.16 |
| **Education**  Illiterate  Can read and write  Primary school level  Secondary school level  Tertiary school  Post-graduate |  |  |  |  | | 1.00  .61  .63  .90  .30  .08 | | .267  .179  .816  .050  .045 | .26  .33  .37  .09  .01 | 1.44  1.22  2.16  .99  .94 |
| **Income**  0-15K  16-30k  31-45k  46-60k  >60k |  |  |  |  | | 1.00  .90  2.00  .82  2.54 | | .712  .162  .774  .093 | .53  .75  .21  .85 | 1.53  5.30  3.12  7.58 |
| **Family structure**  Nuclear family  Three generation family  Extended family |  |  |  |  | | 1.00  2.15  1.46 | | .015  .142 | 1.16  .88 | 4.00  2.42 |
| **Ethnicity**  Gwarri  Hausa  Nupe  Others |  |  |  |  | | 1.00  .86  .95  .76 | | .539  .847  .299 | .53  .58  .46 | 1.39  1.54  1.26 |

**Discussion**

This study provides novel evidence on the relationship between adverse childhood experiences (ACEs) and multimorbidity among older adults in Niger State, North-Central Nigeria. To our knowledge, this study contributes to the limited empirical evidence from this setting on the long-term health implications of cumulative childhood adversity in later life. The findings indicate a high prevalence of ACE exposure in this cohort and show that higher cumulative ACEs are associated with increased odds of multimorbidity among older adults(18).

The distribution of ACEs observed in this study aligns with global evidence indicating that childhood adversity is widespread across diverse populations. International research consistently demonstrates that ACE exposure cuts across racial and ethnic groups, although the magnitude varies by socioeconomic context(18). Importantly, earlier studies have shown that exposure to one ACE increases the likelihood This pattern of co-occurring adversities suggests clustering of childhood exposures, which may increase cumulative risk. Such clustering supports the usefulness of cumulative ACE scores in examining their association with adverse health outcomes in adulthood.(19).

Emotional neglect and exposure to community violence were the most frequently reported ACE domains in this study. Participants reporting these experiences also tended to have a higher number of chronic conditions, suggesting that these specific adversities may be important factors associated with later-life multimorbidity.(19). These findings are consistent with previous studies showing that psychosocial stressors in childhood are significantly associated with chronic disease risk in adulthood. (20). However, variations in the most prevalent ACE domains compared with other Nigerian studies likely reflect regional and sociocultural differences in exposure patterns and reporting practices(21).

The high prevalence of ACEs reported in this cohort must be interpreted cautiously. Although participants were provided sufficient time to recall pre-18 experiences, retrospective reporting may still be influenced by recall bias, particularly among older age groups. Furthermore, because the sample comprised individuals with chronic conditions, the observed distribution of multimorbidity may not fully represent that of the broader population. Educational level may also shape how individuals interpret and report childhood adversity, potentially influencing prevalence estimates (24).

While identifying determinants of ACE exposure was not a primary objective of this study, prior Nigerian research suggests that lower maternal education and lower household wealth were significantly associated with childhood adversity(20). The predominance of low educational attainment and low income among participants in this study may therefore reflect structural vulnerabilities that increase exposure to ACEs and, in turn, are associated with poorer long-term health outcomes These findings suggest a possible intergenerational cycle in which socioeconomic disadvantage increases the likelihood of ACE exposure, which in turn is associated with a greater accumulation of chronic diseases in later life.(20).

The primary objective of this analysis was to examine whether childhood adversity is associated with multimorbidity among older Nigerian adults. The findings show a statistically significant moderate positive correlation between cumulative ACE scores and the number of chronic conditions (r = 0.362, p = 0.01). Hierarchical logistic regression further indicated that cumulative ACE exposure is independently associated with multimorbidity. A clear dose–response pattern was observed, with individuals reporting four or more ACEs showing higher odds of multimorbidity compared with those reporting none, even after adjustment for socio-demographic covariates (Table 5). Although adjustment reduced the magnitude of the association, cumulative ACE exposure remained a significant independent factor associated with multimorbidity.(2,22).

These results are consistent with international evidence showing that cumulative ACE exposure is associated with multimorbidity, functional decline, and premature mortality in adulthood. (2,22). The observed dose–response pattern supports life-course epidemiological models, which propose that early-life adversity has long-term biological and behavioral consequences that shape disease risk across the lifespan. Chronic stress activation, maladaptive coping behaviors, and socioeconomic disadvantage may act as mediating pathways through which ACEs are associated with the clustering of chronic diseases in later life.(23,24).

Overall, the findings indicate that cumulative childhood adversity is significantly associated with multimorbidity among older adults in Nigeria. Integrating ACE screening and trauma-informed approaches into public health policy may improve early identification of high-risk populations and strengthen chronic disease prevention strategies (2,22). A life-course approach that prioritizes early intervention may therefore play a critical role in reducing the long-term burden of multimorbidity in low- and middle-income settings(23,24).

**Limitations**

This study is limited by its cross-sectional design, which precludes causal inference. The hospital-based sample may introduce selection bias and survivor bias, as only older adults who reached ≥60 years were included. Retrospective reporting of ACEs may be affected by recall bias, and residual confounding from unmeasured factors such as early-life socioeconomic conditions and lifestyle exposures cannot be excluded.

**Conclusion**

Cumulative adverse childhood experiences are significantly associated with multimorbidity among older Nigerian adults. These findings underscore the importance of early-life interventions and life-course approaches. Integrating ACE screening and trauma-informed strategies into healthcare and public health policy may improve early identification of high-risk individuals and support strategies to reduce chronic disease accumulation.

**Conflict of interest**

The authors declare no conflict of interest with respect to the research, authorship, and/or publication of this article.

Declarations

**Ethical approval:**

Ethical approval was obtained from the College of Nursing, Midwifery, and Healthcare, Research Ethics Panel, Universty of West London (Ethical Approval No. 1055), and authorization to collect data was obtained from the Research, Ethics, and Publication Committee (REPC) of the Hospitals Management Board, Minna, Niger State of Nigeria. All the participants provided written informed consent before participating in the study.

**Informed Consent Statement**: Participants freely signed their informed consent about 24 h prior to participating in the study, and the individual’s right to withdraw partially or completely was ob‐served

Funding: This research received no external funding.

**References**

1.	Felitti VJ. Adverse childhood experiences and adult health. Acad Pediatr. 2009;9(3):131–2.

2.	Hughes K, Bellis MA, Hardcastle KA, Sethi D, Butchart A, Mikton C, et al. The effect of multiple adverse childhood experiences on health: a systematic review and meta-analysis. Lancet public Heal. 2017;2(8):e356–66.

3.	Senaratne DNS, Thakkar B, Smith BH, Hales TG, Marryat L, Colvin LA. The impact of adverse childhood experiences on multimorbidity: a systematic review and meta-analysis. BMC Med. 2024;22(1):315.

4.	Taylor K, Demakakos P. Adverse childhood experiences and trajectories of multimorbidity in individuals aged over 50: evidence from the English Longitudinal Study of Ageing. Child Abuse Negl. 2024;149:106653.

5.	Shonkoff JP, Garner AS. Committee on psychosocial aspects of child and family health committee on early childhood, adoption, and dependent care section on developmental and behavioral pediatrics the lifelong effects of early childhood adversity and toxic stress. Pediatrics. 2012;129(1):e232–46.

6.	Marengoni A, Angleman S, Melis R, Mangialasche F, Karp A, Garmen A, et al. Aging with multimorbidity: a systematic review of the literature. Ageing Res Rev. 2011;10(4):430–9.

7.	Boyd CM, Darer J, Boult C, Fried LP, Boult L, Wu AW. Clinical practice guidelines and quality of care for older patients with multiple comorbid diseases: implications for pay for performance. Jama. 2005;294(6):716–24.

8.	Organization WH. Global action plan for the prevention and control of noncommunicable diseases 2013-2020. World Health Organization; 2013.

9.	Odunyemi A, Islam MT, Alam K. The financial burden of noncommunicable diseases from out-of-pocket expenditure in sub-Saharan Africa: a scoping review. Health Promot Int. 2024;39(5):daae114.

10.	Gouda HN, Charlson F, Sorsdahl K, Ahmadzada S, Ferrari AJ, Erskine H, et al. Burden of non-communicable diseases in sub-Saharan Africa, 1990–2017: results from the Global Burden of Disease Study 2017. Lancet Glob Heal. 2019;7(10):e1375–87.

11.	Demographic N. National population commission (NPC)[Nigeria] and ICF International. Nigeria: Abuja, Nigeria, and Rockville, Maryland, USA: NPC and ICF International; 2019.

12.	Teicher MH, Samson JA. Annual research review: enduring neurobiological effects of childhood abuse and neglect. J child Psychol psychiatry. 2016;57(3):241–66.

13.	Felitti VJ, Anda RF, Nordenberg D, Williamson DF, Spitz AM, Edwards V, et al. Relationship of childhood abuse and household dysfunction to many of the leading causes of death in adults: The Adverse Childhood Experiences (ACE) Study. Am J Prev Med. 1998;14(4):245–58.

14.	Beard JR, Officer A, De Carvalho IA, Sadana R, Pot AM, Michel JP, et al. The World report on ageing and health: a policy framework for healthy ageing. Lancet. 2016;387(10033):2145–54.

15.	Ahmed A, Khan HTA, Lawal M. The Prevalence, Pattern, and Burden of Multimorbidity Among Older Adults in Niger State, Northern Nigeria. Illness, Cris Loss. 2025;33(3):684–701.

16.	Ahmed A, Khan HTA, Lawal M. Patients’ satisfaction with healthcare services among older people with multimorbidity: Subnational gender perspective. J Popul Ageing. 2024;1–14.

17.	Ahmed A, Khan HTA, Lawal M. Effective hospital care delivery model for older people in Nigeria with multimorbidity: recommendations for practice. In: Healthcare. MDPI; 2022. p. 1268.

18.	Bethell CD, Davis MB, Gombojav N, Stumbo S, Powers K. Issue brief: Adverse childhood experiences among US children. Child Adolesc Heal Meas Initiat Johns Hopkins Bloom Sch Public Heal. 2017;

19.	Vásquez E, Quiñones A, Ramirez S, Udo T. Association between adverse childhood events and multimorbidity in a racial and ethnic diverse sample of middle-aged and older adults. Innov aging. 2019;3(2):igz016.

20.	Salawu MM, Owoaje ET. Prevalence and predictors of adverse childhood experiences among youths in rural communities of Oyo State, South-west, Nigeria. J Community Med Prim Heal Care. 2020;32(2):27–41.

21.	Vannorsdall TD, Munro CA. The link between childhood adversity and late-life mental health: evidence for the influence of early-life experiences or illusory correlations? Int Psychogeriatrics. 2017;29(3):357–8.

22.	Brown DW, Anda RF, Tiemeier H, Felitti VJ, Edwards VJ, Croft JB, et al. Adverse childhood experiences and the risk of premature mortality. Am J Prev Med. 2009;37(5):389–96.

23.	Bethell C, Jones J, Gombojav N, Linkenbach J, Sege R. Positive childhood experiences and adult mental and relational health in a statewide sample: Associations across adverse childhood experiences levels. JAMA Pediatr. 2019;173(11):e193007.

24.	Lacey RE, Minnis H. Practitioner review: twenty years of research with adverse childhood experience scores–advantages, disadvantages and applications to practice. J Child Psychol Psychiatry. 2020;61(2):116–30.
