# Supplementary material for: The impact of adverse childhood experiences on the development of multimorbidity in older adults in Nigeria
Source: PLoS One. 2026 Sep 25;21(9):e0358944. doi: 10.1371/journal.pone.0358944 (PMC13614559; doi:10.1371/journal.pone.0358944)
Supplement: Supplementary file 7 [file pone.0358944.s008.docx]

**Detailed Response to Editor and Reviewer Comments on Revised Manuscript**

Dear Editor,

We are grateful for your careful re-evaluation of our revised manuscript and for the constructive and insightful comments provided. We appreciate your initiative in reviewing the manuscript in the absence of the second reviewer and for highlighting areas requiring further clarification. We have addressed all comments thoroughly and revised the manuscript accordingly to enhance its methodological transparency, analytical rigor, and interpretative balance. Our detailed responses are presented below.

1. **Magnitude and Precision of the Reported Odds Ratios**

We acknowledge the concern regarding the magnitude and imprecision of the crude (OR = 67.14; 95% CI: 9.25–487.04) and adjusted odds ratios (OR = 15.58; 95% CI: 1.33–182.21). In the revised manuscript, we have:

- Explicitly emphasized that while the association between ACEs and multimorbidity is statistically significant, the effect size should be interpreted with caution due to wide confidence intervals.
- Added text in the section explaining that the wide intervals likely reflect sparse data in some exposure categories and variability in responses, limiting precision **(page 10, line 314 to 316).**

2. **Further Elaboration on Recall Bias**

We appreciate the recognition of our acknowledgment of recall bias and have expanded this section further. Specifically, we now clarify that:

- Retrospective reporting of adverse childhood experiences (ACEs), particularly among older participants, is susceptible to differential and non-differential misclassification.
- Recall bias may lead to systematic over-reporting (due to current health status influencing recollection) or under-reporting (due to memory decay or social desirability), thereby potentially biasing effect estimates in either direction.
- This limitation has been more explicitly articulated in the manuscript to ensure a balanced interpretation of findings.

**(Page 13, 399 to 408)**

3. **Model Diagnostics: Goodness-of-Fit and Multicollinearity**

In response to the reviewer’s methodological query, we have strengthened the Methods and Results sections as follows:

- We have explicitly described the procedures used to assess model adequacy, including the Hosmer–Lemeshow goodness-of-fit test, which indicated acceptable model calibration.
- We assessed multicollinearity using Variance Inflation Factors (VIFs), all of which were within acceptable thresholds, suggesting no evidence of problematic collinearity among covariates.

**(Page 5, line 249 to 252)**

4. Attenuation of the Odds Ratio Following Multivariable Adjustment
We agree that the substantial reduction in the odds ratio following adjustment warrants further explanation. Accordingly:

- We have expanded the Discussion to clarify that the attenuation from OR = 67.14 to OR = 15.58 reflects confounding by socio-demographic variables included in the multivariable model.
- Specifically, factors such as age, educational attainment, and socioeconomic status partially accounted for the crude association, indicating that these variables are important determinants of both ACE exposure and multimorbidity.
- Where analytically feasible, we have indicated the covariate(s) that contributed most to the reduction in the effect estimate, thereby providing a clearer interpretation of the independent association between ACE exposure and multimorbidity.
- This additional discussion strengthens the causal interpretability of our findings while maintaining appropriate caution.
- **(page 10, line 314 to 316).**

5. Inclusion of Methodological Details as Supplementary Material
In line with your recommendation, we have included a comprehensive description of the study methodology, rather than referring readers to prior publications alone. This includes:

- Sampling procedures
- Measurement of ACEs and multimorbidity
- Data collection protocols
- Analytical framework

**(Pages 3 to 4, lines 174 to 189)**

This addition enhances the manuscript’s transparency and allows for independent appraisal of the study design and execution.

In summary, we have carefully addressed all comments and believe that the revisions have substantially improved the clarity, methodological robustness, and interpretative balance of the manuscript. We are grateful for the opportunity to refine our work and for the valuable guidance provided.

Thank you for your consideration.

Yours sincerely,
Nestor Asiamah
On behalf of all authors
