# Supplementary material for: The impact of adverse childhood experiences on the development of multimorbidity in older adults in Nigeria
Source: PLoS One. 2026 Sep 25;21(9):e0358944. doi: 10.1371/journal.pone.0358944 (PMC13614559; doi:10.1371/journal.pone.0358944)
Supplement: Peer review history

# Peer review: Decision Letter 0

**DOI:** 10.1371/journal.pone.0358944.r001

**Authors:** Mithun Sikdar

9 Feb 2026

Dear Dr. Asiamah,

Thank you for submitting your manuscript to PLOS ONE. After careful consideration, we feel that it has merit but does not fully meet PLOS ONE’s publication criteria as it currently stands. Therefore, we invite you to submit a revised version of the manuscript that addresses the points raised during the review process.

Please submit your revised manuscript by Mar 26 2026 11:59PM. If you will need more time than this to complete your revisions, please reply to this message or contact the journal office at plosone@plos.org. When you're ready to submit your revision, log on to https://www.editorialmanager.com/pone/ and select the 'Submissions Needing Revision' folder to locate your manuscript file.

- A letter that responds to each point raised by the academic editor and reviewer(s). You should upload this letter as a separate file labeled 'Response to Reviewers'.
- A marked-up copy of your manuscript that highlights changes made to the original version. You should upload this as a separate file labeled 'Revised Manuscript with Track Changes'.
- An unmarked version of your revised paper without tracked changes. You should upload this as a separate file labeled 'Manuscript'.

If you would like to make changes to your financial disclosure, please include your updated statement in your cover letter. Guidelines for resubmitting your figure files are available below the reviewer comments at the end of this letter.

If applicable, we recommend that you deposit your laboratory protocols in protocols.io to enhance the reproducibility of your results. Protocols.io assigns your protocol its own identifier (DOI) so that it can be cited independently in the future. For instructions see: https://journals.plos.org/plosone/s/submission-guidelines#loc-laboratory-protocols. Additionally, PLOS ONE offers an option for publishing peer-reviewed Lab Protocol articles, which describe protocols hosted on protocols.io. Read more information on sharing protocols at https://plos.org/protocols?utm_medium=editorial-email&utm_source=authorletters&utm_campaign=protocols.

We look forward to receiving your revised manuscript.

Kind regards,

Mithun Sikdar

Academic Editor

PLOS One

Journal Requirements:

When submitting your revision, we need you to address these additional requirements.

1.Please ensure that your manuscript meets PLOS ONE's style requirements, including those for file naming. The PLOS ONE style templates can be found at

https://journals.plos.org/plosone/s/file?id=wjVg/PLOSOne_formatting_sample_main_body.pdf and

https://journals.plos.org/plosone/s/file?id=ba62/PLOSOne_formatting_sample_title_authors_affiliations.pdf

2. Please include captions for your Supporting Information files at the end of your manuscript, and update any in-text citations to match accordingly. Please see our Supporting Information guidelines for more information: http://journals.plos.org/plosone/s/supporting-information.

3. We are unable to open your Supporting Information file “Data.sav and PONE-D-25-04688 _proof.pdf” Please kindly revise as necessary and re-upload.

4. If the reviewer comments include a recommendation to cite specific previously published works, please review and evaluate these publications to determine whether they are relevant and should be cited. There is no requirement to cite these works unless the editor has indicated otherwise.

Additional Editor Comments:

Considering the reviewers’ comments, the paper requires major revision. I believe the authors will make the necessary effort to revise the manuscript in accordance with the reviewers’ suggestions.

[Note: HTML markup is below. Please do not edit.]

Reviewer's Responses to Questions

**Comments to the Author**

1. Is the manuscript technically sound, and do the data support the conclusions?

Reviewer #1: Yes

Reviewer #2: Yes

**********

2. Has the statistical analysis been performed appropriately and rigorously?

Reviewer #1: No

Reviewer #2: Yes

**********

3. Have the authors made all data underlying the findings in their manuscript fully available?

The PLOS Data policy

Reviewer #1: Yes

Reviewer #2: Yes

**********

4. Is the manuscript presented in an intelligible fashion and written in standard English?

Reviewer #1: Yes

Reviewer #2: Yes

**********

Reviewer #1: This manuscript addresses an important topic and offers useful evidence on the association between ACEs and multimorbidity among older adults in Nigeria, but several issues require attention before publication. The writing, while understandable, contains frequent grammatical and structural problems that need editing for clarity and academic tone. Methodological limitations, particularly the cross-sectional hospital-based design, categorization of count variables, and very large, imprecise odds ratios as well as limit causal interpretation and warrant more cautious conclusions. The manuscript also lacks a formal Data Availability Statement, and details on missing data handling and model diagnostics are insufficient. Ethics approval appears appropriate, and no concerns about misconduct arise, but the introduction, methods, and discussion would benefit from clearer organization and more consistent reporting. With revisions to improve language, transparency, analytical justification, and framing of conclusions, the study could make a meaningful contribution to life-course research. A full review has been uploaded for your consideration

Reviewer #2: 1.You can state that it is a clinic geriatric study because it is studied on the OPD hospitals not on the community of Nigeria or not the randomly sampled healthy older adults.

2.Given that socioeconomic conditions in Nigeria are deeply embedded within cultural practices and extended family systems, certain ACE items related to neglect, household instability, or economic hardship may be culturally contextual rather than purely adverse. This raises the possibility of some ACE indicators may be subject to bias or overestimation of ACE exposure.

3.Author can mention that they have used the binary version for calculating the ACE cumulative scores.

4.While this study employs the standardized ACE framework like Neglect, abuse, violence etc. ACE should also be understood localized ACE construct like food insecurity and child labor as a salient childhood stressor which are not properly captured in ACE questionnaire.

4.Glaucoma, Cataract, Obesity, Liver Cirrhosis, arthritis, Osteoporosis though falls under expanded chronic diseases criteria of WHO. Does it also fall under PURE defined chronic diseases?

**********

what does this mean?). If published, this will include your full peer review and any attached files.

If you choose “no”, your identity will remain anonymous but your review may still be made public.

**Do you want your identity to be public for this peer review?** For information about this choice, including consent withdrawal, please see our Privacy Policy

Reviewer #1: No

Reviewer #2: **Yes:**  TANUSHREE BARMAN

**********

[NOTE: If reviewer comments were submitted as an attachment file, they will be attached to this email and accessible via the submission site. Please log into your account, locate the manuscript record, and check for the action link "View Attachments". If this link does not appear, there are no attachment files.]

To ensure your figures meet our technical requirements, please review our figure guidelines: https://journals.plos.org/plosone/s/figures

You may also use PLOS’s free figure tool, NAAS, to help you prepare publication quality figures: https://journals.plos.org/plosone/s/figures#loc-tools-for-figure-preparation.

NAAS will assess whether your figures meet our technical requirements by comparing each figure against our figure specifications.

Attachment Submitted filename: Manuscript Review (PONE-D-25-20171)..pdf

Attachment Submitted filename: Manuscript Revised final.docx

# Author comment: Author response to Decision Letter 1

**DOI:** 10.1371/journal.pone.0358944.r002

2 Mar 2026

The response to reviewers and editor is attached

Attachment Submitted filename: AUTHORS RESPONSE TO REVIEWERS.docx

# Peer review: Decision Letter 1

**DOI:** 10.1371/journal.pone.0358944.r003

**Authors:** Mithun Sikdar

8 May 2026

Dear Dr. Asiamah,

Thank you for submitting your manuscript to PLOS ONE. After careful consideration, we feel that it has merit but does not fully meet PLOS ONE’s publication criteria as it currently stands. Therefore, we invite you to submit a revised version of the manuscript that addresses the points raised during the review process.

Dear Authors,

I appreciate the effort you have put into improving the manuscript; however, it still requires further revision.

Page 3:

Please elaborate the larger part of the study on which the present study is based.

Please revise the abstract to reflect a clearer scientific orientation. The use of a “chronological approach” needs clarification, does the study incorporate a longitudinal design to determine the onset and progression of multimorbidity? It appears that such an approach has not been employed. Rather, the study seems to rely on current prevalence of multimorbidity alongside retrospective assessment of adverse childhood experiences (ACEs), which would more appropriately classify it as an analytical observational study. Please revise the abstract.

Additionally, the manuscript requires careful linguistic refinement to meet the standards of a reputed journal such as *PLOS ONE* .

Several sentences are overly long and at times difficult to follow, affecting overall clarity and readability. For example:

Page 1

“Face-to-face data was collected from a cross-section of 734 patients aged 60 years and over with 2 or more chronic diseases who visit for routine outpatient care and consented to participate in the study”.

Page 8

“It has been found in Table 3 that ACEs is strongly associated with multimorbidity as indicated by Chi-squared test (<img height="21" src="data:image/png;base64,iVBORw0KGgoAAAANSUhEUgAAABoAAAAfCAMAAADOWS1PAAAAAXNSR0IArs4c6QAAAGNQTFRFAAAAAAAAAAA6AABmADpmADqQAGa2OgAAOjoAOjo6Oma2OpDbZgAAZpC2ZrbbZrb/kDoAkDpmkLbbkNv/tmYAtmY6tpA6tpBmttv/tv//25A627Zm2////7Zm/9uQ//+2///b5CQKPwAAAAF0Uk5TAEDm2GYAAAAJcEhZcwAAFiUAABYlAUlSJPAAAAAZdEVYdFNvZnR3YXJlAE1pY3Jvc29mdCBPZmZpY2V/7TVxAAAAr0lEQVQ4T8WS3Q7CIAyFqWxDHW6o9WdsDN7/KdfihcnSXhrPBSGUcr6eYMwfVV5HgOYtESAMZnV2EmrY0SEe7ho4il18O3tulVRCu2gV7bmCVkTf080OztUcyD3ByHVejJkvS4VF6BZTArC+cyXaJhC5Vjcq4ZDNEJRssj9Vd0Fk3ivRJMYWtTqtlH37lLtKsFOUs0aCi2LYD/aJ0lSx5pOgL9f9f/gkSYM1t19/8Q1ptQixyyI7JwAAAABJRU5ErkJggg==" width="17" />=116.07, p<.001) which means that multimorbidity in old age is associated with ACEs. Older adults who had experienced 4 or more ACEs are found to have suffered more with 3+ diseases compared to older people with <3 diseases. Other selected covariates such as age, gender, marital status, occupation, education, income, family structure are also found to be statistically associated with multimorbidity. However, ethnicity is found to be not statistically associated with multimorbidity in the study.”

The method part needs elaboration.

I have appended the revisions suggested by the reviewers. They have recommended major revisions before the manuscript can be considered suitable for publication.

**Reviewer 1:**

Generally, the manuscript has improved; however, several critical issues remain that need to be addressed before it can be considered for publication.

Firstly: the use of causal language is still evident and is not appropriate for a cross-sectional study design.

Phrases such as “predicts multimorbidity” and “demonstrating a clear dose-response relationship” imply causality and should be revised to reflect associations only (example, “is associated with,” “is linked to,” or “shows a dose–response pattern”).

Secondly:

- the Methods section remains insufficiently detailed. While it is noted that this study is part of a larger study, the manuscript must be self-contained, as readers may not have access to the parent study.

A clear and explicit description of the study design (e.g., cross-sectional hospital-based study) should be provided upfront. Additionally, key methodological details are still missing or unclear, including the sampling frame, inclusion and exclusion criteria, ACE-IQ scoring approach, regression model specification, handling of missing data, and clarification of reliability measures such as Cronbach’s alpha.

The current description still does not adequately address concerns.

Thirdly:

- concerns regarding the logistic regression analysis have not been addressed.

- The reported large odds ratios, wide confidence intervals, and potential model instability require explanation.

- This is a significant issue that should be discussed, with possible reasons such as sparse data bias, small subgroup sizes, or effects of variable categorization clearly articulated in the Discussion.

Fourthly:

- the justification for using a cutoff of three or more diseases remains unclear.

- This threshold should be supported by appropriate references or a clear rationale (example, clinical relevance or distribution-based justification).

Fifthly:

- the Results section still contains interpretative language.

- Statements such as “strongly associated” and “found to have suffered more” should be revised to ensure that results are presented in a strictly objective and descriptive manner, with interpretation reserved for the Discussion.

- Additionally, there are instances of inappropriate or overstated claims in the manuscript. For example, statements such as “To the best of our knowledge, this is the first study that analyses the impact of ACEs on the health of older adults in Niger state north-central Nigeria” are not suitable for scientific writing unless they are clearly substantiated by a comprehensive and systematic literature search. Such claims are often difficult to verify and may be perceived as overstated. It is recommended to either remove such statements or rephrase them more cautiously (example, “This study contributes to the limited body of literature on…”).

Finally:

- the discussion of bias and limitations is still underdeveloped.

- Important sources of bias, including selection bias, survivor bias, recall bias, and residual confounding, should be more thoroughly addressed to strengthen the rigor and transparency of the manuscript.

Overall, while revisions have been made, substantial improvements are still required.

Generally, the manuscript has improved; however, several critical issues remain that need to be addressed before it can be considered for publication.

Firstly:

- the use of causal language is still evident and is not appropriate for a cross-sectional study design.

Phrases such as “predicts multimorbidity” and “demonstrating a clear dose–response relationship” imply causality and should be revised to reflect associations only (example, “is associated with,” “is linked to,” or “shows a dose–response pattern”).

Secondly:

- the Methods section remains insufficiently detailed. While it is noted that this study is part of a larger study, the manuscript must be self-contained, as readers may not have access to the parent study.

A clear and explicit description of the study design (e.g., cross-sectional hospital-based study) should be provided upfront. Additionally, key methodological details are still missing or unclear, including the sampling frame, inclusion and exclusion criteria, ACE-IQ scoring approach, regression model specification, handling of missing data, and clarification of reliability measures such as Cronbach’s alpha.

The current description still does not adequately address concerns.

Thirdly:

- concerns regarding the logistic regression analysis have not been addressed.

- The reported large odds ratios, wide confidence intervals, and potential model instability require explanation.

- This is a significant issue that should be discussed, with possible reasons such as sparse data bias, small subgroup sizes, or effects of variable categorization clearly articulated in the Discussion.

Fourthly:

- the justification for using a cutoff of three or more diseases remains unclear.

- This threshold should be supported by appropriate references or a clear rationale (example, clinical relevance or distribution-based justification).

Fifthly:

- the Results section still contains interpretative language.

- Statements such as “strongly associated” and “found to have suffered more” should be revised to ensure that results are presented in a strictly objective and descriptive manner, with interpretation reserved for the Discussion.

- Additionally, there are instances of inappropriate or overstated claims in the manuscript. For example, statements such as “To the best of our knowledge, this is the first study that analyses the impact of ACEs on the health of older adults in Niger state north-central Nigeria” are not suitable for scientific writing unless they are clearly substantiated by a comprehensive and systematic literature search. Such claims are often difficult to verify and may be perceived as overstated. It is recommended to either remove such statements or rephrase them more cautiously (example, “This study contributes to the limited body of literature on…”).

Finally:

- the discussion of bias and limitations is still underdeveloped.

- Important sources of bias, including selection bias, survivor bias, recall bias, and residual confounding, should be more thoroughly addressed to strengthen the rigor and transparency of the manuscript.

Overall, while revisions have been made, substantial improvements are still required.

**Reviewer 2**

Justify why there is a big difference in the odds ratio. The odds ratio between 0 morbidity and 4 or more morbidities is very high.

==============================

Please submit your revised manuscript by Jun 22 2026 11:59PM. If you will need more time than this to complete your revisions, please reply to this message or contact the journal office at plosone@plos.org. When you're ready to submit your revision, log on to https://www.editorialmanager.com/pone/ and select the 'Submissions Needing Revision' folder to locate your manuscript file.

Please include the following items when submitting your revised manuscript:

- A letter that responds to each point raised by the academic editor and reviewer(s). You should upload this letter as a separate file labeled 'Response to Reviewers'.
- A marked-up copy of your manuscript that highlights changes made to the original version. You should upload this as a separate file labeled 'Revised Manuscript with Track Changes'.
- An unmarked version of your revised paper without tracked changes. You should upload this as a separate file labeled 'Manuscript'.

If you would like to make changes to your financial disclosure, please include your updated statement in your cover letter. Guidelines for resubmitting your figure files are available below the reviewer comments at the end of this letter.

If applicable, we recommend that you deposit your laboratory protocols in protocols.io to enhance the reproducibility of your results. Protocols.io assigns your protocol its own identifier (DOI) so that it can be cited independently in the future. For instructions see: https://journals.plos.org/plosone/s/submission-guidelines#loc-laboratory-protocols. Additionally, PLOS ONE offers an option for publishing peer-reviewed Lab Protocol articles, which describe protocols hosted on protocols.io. Read more information on sharing protocols at https://plos.org/protocols?utm_medium=editorial-email&utm_source=authorletters&utm_campaign=protocols.

As the corresponding author, your ORCID iD is verified in the submission system and will appear in the published article. PLOS supports the use of ORCID, and we encourage all coauthors to register for an ORCID iD and use it as well. Please encourage your coauthors to verify their ORCID iD within the submission system before final acceptance, as unverified ORCID iDs will not appear in the published article. *Only* the individual author can complete the verification step; PLOS staff *cannot* verify ORCID iDs on behalf of authors.

We look forward to receiving your revised manuscript.

Kind regards,

Mithun Sikdar

Academic Editor

PLOS One

**Journal Requirements:**

If the reviewer comments include a recommendation to cite specific previously published works, please review and evaluate these publications to determine whether they are relevant and should be cited. There is no requirement to cite these works unless the editor has indicated otherwise.

[Note: HTML markup is below. Please do not edit.]

Reviewers' comments:

Reviewer's Responses to Questions

**Comments to the Author**

Reviewer #1: (No Response)

Reviewer #2: All comments have been addressed

**********

2. Is the manuscript technically sound, and do the data support the conclusions?

Reviewer #1: Partly

Reviewer #2: Yes

**********

3. Has the statistical analysis been performed appropriately and rigorously?

Reviewer #1: Yes

Reviewer #2: No

**********

4. Have the authors made all data underlying the findings in their manuscript fully available?

The PLOS Data policy

Reviewer #1: Yes

Reviewer #2: Yes

**********

5. Is the manuscript presented in an intelligible fashion and written in standard English?

Reviewer #1: Yes

Reviewer #2: Yes

**********

Reviewer #1: Review of PONE-D-25-20171R1

Generally, the manuscript has improved; however, several critical issues remain that need to be addressed before it can be considered for publication.

Firstly:

- the use of causal language is still evident and is not appropriate for a cross-sectional study design.

Phrases such as “predicts multimorbidity” and “demonstrating a clear dose–response relationship” imply causality and should be revised to reflect associations only (example, “is associated with,” “is linked to,” or “shows a dose–response pattern”).

Secondly:

- the Methods section remains insufficiently detailed. While it is noted that this study is part of a larger study, the manuscript must be self-contained, as readers may not have access to the parent study.

A clear and explicit description of the study design (e.g., cross-sectional hospital-based study) should be provided upfront. Additionally, key methodological details are still missing or unclear, including the sampling frame, inclusion and exclusion criteria, ACE-IQ scoring approach, regression model specification, handling of missing data, and clarification of reliability measures such as Cronbach’s alpha.

The current description still does not adequately address concerns.

Thirdly:

- concerns regarding the logistic regression analysis have not been addressed.

- The reported large odds ratios, wide confidence intervals, and potential model instability require explanation.

- This is a significant issue that should be discussed, with possible reasons such as sparse data bias, small subgroup sizes, or effects of variable categorization clearly articulated in the Discussion.

Fourthly:

- the justification for using a cutoff of three or more diseases remains unclear.

- This threshold should be supported by appropriate references or a clear rationale (example, clinical relevance or distribution-based justification).

Fifthly:

- the Results section still contains interpretative language.

- Statements such as “strongly associated” and “found to have suffered more” should be revised to ensure that results are presented in a strictly objective and descriptive manner, with interpretation reserved for the Discussion.

- Additionally, there are instances of inappropriate or overstated claims in the manuscript. For example, statements such as “To the best of our knowledge, this is the first study that analyses the impact of ACEs on the health of older adults in Niger state north-central Nigeria” are not suitable for scientific writing unless they are clearly substantiated by a comprehensive and systematic literature search. Such claims are often difficult to verify and may be perceived as overstated. It is recommended to either remove such statements or rephrase them more cautiously (example, “This study contributes to the limited body of literature on…”).

Finally:

- the discussion of bias and limitations is still underdeveloped.

- Important sources of bias, including selection bias, survivor bias, recall bias, and residual confounding, should be more thoroughly addressed to strengthen the rigor and transparency of the manuscript.

Overall, while revisions have been made, substantial improvements are still required.

Reviewer #2: Justify why there is a big difference in the odds ratio. The odds ratio between 0 morbidity and 4 or more morbidities is very high.

**********

what does this mean?). If published, this will include your full peer review and any attached files.

If you choose “no”, your identity will remain anonymous but your review may still be made public.

**Do you want your identity to be public for this peer review?** For information about this choice, including consent withdrawal, please see our Privacy Policy

Reviewer #1: No

Reviewer #2: **Yes:**  Tanushree Barman

**********

[NOTE: If reviewer comments were submitted as an attachment file, they will be attached to this email and accessible via the submission site. Please log into your account, locate the manuscript record, and check for the action link "View Attachments". If this link does not appear, there are no attachment files.]

To ensure your figures meet our technical requirements, please review our figure guidelines: https://journals.plos.org/plosone/s/figures

You may also use PLOS’s free figure tool, NAAS, to help you prepare publication quality figures: https://journals.plos.org/plosone/s/figures#loc-tools-for-figure-preparation.

NAAS will assess whether your figures meet our technical requirements by comparing each figure against our figure specifications.

Attachment Submitted filename: Manuscript.docx

# Author comment: Author response to Decision Letter 2

**DOI:** 10.1371/journal.pone.0358944.r004

18 May 2026

The response to reviewers is attached as a file

Attachment Submitted filename: Response to Editor and Reviewers.docx

# Peer review: Decision Letter 2

**DOI:** 10.1371/journal.pone.0358944.r005

**Authors:** Mithun Sikdar

17 Jul 2026

Dear Dr. Asiamah,

Thank you for submitting your manuscript to PLOS ONE. After careful consideration, we feel that it has merit but does not fully meet PLOS ONE’s publication criteria as it currently stands. Therefore, we invite you to submit a revised version of the manuscript that addresses the points raised during the review process.

Please submit your revised manuscript by Aug 31 2026 11:59PM. If you will need more time than this to complete your revisions, please reply to this message or contact the journal office at plosone@plos.org. When you're ready to submit your revision, log on to https://www.editorialmanager.com/pone/ and select the 'Submissions Needing Revision' folder to locate your manuscript file.

- A letter that responds to each point raised by the academic editor and reviewer(s). You should upload this letter as a separate file labeled 'Response to Reviewers'.
- A marked-up copy of your manuscript that highlights changes made to the original version. You should upload this as a separate file labeled 'Revised Manuscript with Track Changes'.
- An unmarked version of your revised paper without tracked changes. You should upload this as a separate file labeled 'Manuscript'.

If you would like to make changes to your financial disclosure, please include your updated statement in your cover letter. Guidelines for resubmitting your figure files are available below the reviewer comments at the end of this letter.

If applicable, we recommend that you deposit your laboratory protocols in protocols.io to enhance the reproducibility of your results. Protocols.io assigns your protocol its own identifier (DOI) so that it can be cited independently in the future. For instructions see: https://journals.plos.org/plosone/s/submission-guidelines#loc-laboratory-protocols. Additionally, PLOS ONE offers an option for publishing peer-reviewed Lab Protocol articles, which describe protocols hosted on protocols.io. Read more information on sharing protocols at https://plos.org/protocols?utm_medium=editorial-email&utm_source=authorletters&utm_campaign=protocols.

As the corresponding author, your ORCID iD is verified in the submission system and will appear in the published article. PLOS supports the use of ORCID, and we encourage all coauthors to register for an ORCID iD and use it as well. Please encourage your coauthors to verify their ORCID iD within the submission system before final acceptance, as unverified ORCID iDs will not appear in the published article. *Only* the individual author can complete the verification step; PLOS staff *cannot* verify ORCID iDs on behalf of authors.

We look forward to receiving your revised manuscript.

Kind regards,

Mithun Sikdar

Academic Editor

PLOS One

Journal Requirements:

If the reviewer comments include a recommendation to cite specific previously published works, please review and evaluate these publications to determine whether they are relevant and should be cited. There is no requirement to cite these works unless the editor has indicated otherwise.

Please review your reference list to ensure that it is complete and correct. If you have cited papers that have been retracted, please include the rationale for doing so in the manuscript text, or remove these references and replace them with relevant current references. Any changes to the reference list should be mentioned in the rebuttal letter that accompanies your revised manuscript. If you need to cite a retracted article, indicate the article’s retracted status in the References list and also include a citation and full reference for the retraction notice.

Additional Editor Comments:

The revised manuscript is substantially improved, with a much clearer and more coherent flow of writing. Although the second reviewer was not available to evaluate the revised manuscript, I took the initiative to review the entire revised version and suggest the minor revisions that both the first reviewer and I considered necessary. The crude OR of 67.14 is extremely high and the confidence interval is extremely wide, spanning from 9.25 to 487.04. The adjustment substantially reduced the OR from 67 to 15.58. The findings support a significant association between ACEs and multimorbidity, but the reported odds ratios, particularly the crude OR of 67.14 and the adjusted OR with a 95% CI of 1.33-182.21 are too imprecise to support a confident claim about the true effect size. The association is likely real, but the magnitude should be interpreted with considerable caution.

It is good that the authors have accepted the limitation of recall bias (The high prevalence of ACEs reported in this cohort must be interpreted cautiously. Although participants were provided sufficient time to recall pre-18 experiences, retrospective reporting may still be influenced by recall bias, particularly among older age groups). How this recall bias can influence the results can also be little elaborated.

The reviewer raised two important methodological concerns that should be addressed in the revised manuscript.

First, the reviewer requested clarification regarding model diagnostics, stating:

"Model fit was assessed using VIF or the Hosmer-Lemeshow goodness-of-fit test? Please include the results of the Hosmer-Lemeshow test and the variance inflation factors (VIFs), either in the main text or as supplementary material."

Accordingly, the manuscript should clearly describe the procedures used to assess model fit and multicollinearity. If these analyses were performed, the corresponding results should be presented either in the main manuscript or as supplementary material.

Second, the reviewer commented on the marked change in the odds ratio following multivariable adjustment:

"The substantial reduction in the odds ratio after multivariable adjustment (from 67.14 to 15.58) needs further discussion. While the adjusted association remains statistically significant, the manuscript does not explain the likely reasons for this reduction. A brief discussion of the role of confounding by the adjusted socio-demographic variables would help to better interpret the effect of ACE exposure. If possible, the authors may also indicate which covariates contributed most to the reduction in the effect estimate." This point warrants a more detailed discussion. The authors should explain that the attenuation of the odds ratio following multivariable adjustment is indicative of confounding by the socio-demographic variables included in the model. If feasible, they should also identify the covariate(s) that contributed most to the reduction in the effect estimate, thereby providing a clearer interpretation of the independent association between ACE exposure and the study outcome.

May I request the authors to include the methodology as supplementary material rather than simply referring readers to previous publications for methodological details?

I believe the authors will be able to address these minor revisions before the manuscript is published in its present form.

[Note: HTML markup is below. Please do not edit.]

Reviewers' comments:

Reviewer's Responses to Questions

**Comments to the Author**

Reviewer #2: All comments have been addressed

**********

2. Is the manuscript technically sound, and do the data support the conclusions?

Reviewer #2: Yes

**********

3. Has the statistical analysis been performed appropriately and rigorously?

Reviewer #2: No

**********

4. Have the authors made all data underlying the findings in their manuscript fully available?

The PLOS Data policy

Reviewer #2: Yes

**********

5. Is the manuscript presented in an intelligible fashion and written in standard English?

Reviewer #2: Yes

**********

Reviewer #2: 1.Model fit was assessed using VIF OR Hosmer–Lemeshow goodness-of-fit test? Please paste these results (Hosmer–Lemeshow test and (VIFs)) either in the main text or as supplementary material. Both the Pdf and word file have different versions.

2.In the statistical analysis section-The substantial reduction in the odds ratio after multivariable adjustment (from 67.14 to 15.58) needs further discussion. While the adjusted association remains statistically significant, the manuscript does not explain the likely reasons for this reduction. A brief discussion of the role of confounding by the adjusted socio-demographic variables would help to interpret better the effect of ACE exposure. If possible, the authors may also indicate which covariates contributed most to the reduction in the effect estimate.

**********

what does this mean?). If published, this will include your full peer review and any attached files.

If you choose “no”, your identity will remain anonymous but your review may still be made public.

**Do you want your identity to be public for this peer review?** For information about this choice, including consent withdrawal, please see our Privacy Policy

Reviewer #2: **Yes:**  TANUSHREE BARMAN

**********

[NOTE: If reviewer comments were submitted as an attachment file, they will be attached to this email and accessible via the submission site. Please log into your account, locate the manuscript record, and check for the action link "View Attachments". If this link does not appear, there are no attachment files.]

To ensure your figures meet our technical requirements, please review our figure guidelines: https://journals.plos.org/plosone/s/figures

You may also use PLOS’s free figure tool, NAAS, to help you prepare publication quality figures: https://journals.plos.org/plosone/s/figures#loc-tools-for-figure-preparation.

NAAS will assess whether your figures meet our technical requirements by comparing each figure against our figure specifications.

Attachment Submitted filename: Manuscript (7) R.docx

# Author comment: Author response to Decision Letter 3

**DOI:** 10.1371/journal.pone.0358944.r006

19 Aug 2026

Dear Editor,

We are grateful for your careful re-evaluation of our revised manuscript and for the constructive and insightful comments provided. We appreciate your initiative in reviewing the manuscript in the absence of the second reviewer and for highlighting areas requiring further clarification. We have addressed all comments thoroughly and revised the manuscript accordingly to enhance its methodological transparency, analytical rigor, and interpretative balance. Our detailed responses are presented below.

1. Magnitude and Precision of the Reported Odds Ratios

We acknowledge the concern regarding the magnitude and imprecision of the crude (OR = 67.14; 95% CI: 9.25–487.04) and adjusted odds ratios (OR = 15.58; 95% CI: 1.33–182.21). In the revised manuscript, we have:

• Explicitly emphasized that while the association between ACEs and multimorbidity is statistically significant, the effect size should be interpreted with caution due to wide confidence intervals.

• Added text in the section explaining that the wide intervals likely reflect sparse data in some exposure categories and variability in responses, limiting precision (page 10, line 314 to 316).

2. Further Elaboration on Recall Bias

We appreciate the recognition of our acknowledgment of recall bias and have expanded this section further. Specifically, we now clarify that:

• Retrospective reporting of adverse childhood experiences (ACEs), particularly among older participants, is susceptible to differential and non-differential misclassification.

• Recall bias may lead to systematic over-reporting (due to current health status influencing recollection) or under-reporting (due to memory decay or social desirability), thereby potentially biasing effect estimates in either direction.

• This limitation has been more explicitly articulated in the manuscript to ensure a balanced interpretation of findings.

(Page 13, 399 to 408)

3. Model Diagnostics: Goodness-of-Fit and Multicollinearity

In response to the reviewer’s methodological query, we have strengthened the Methods and Results sections as follows:

• We have explicitly described the procedures used to assess model adequacy, including the Hosmer–Lemeshow goodness-of-fit test, which indicated acceptable model calibration.

• We assessed multicollinearity using Variance Inflation Factors (VIFs), all of which were within acceptable thresholds, suggesting no evidence of problematic collinearity among covariates.

(Page 5, line 249 to 252)

4. Attenuation of the Odds Ratio Following Multivariable Adjustment

We agree that the substantial reduction in the odds ratio following adjustment warrants further explanation. Accordingly:

• We have expanded the Discussion to clarify that the attenuation from OR = 67.14 to OR = 15.58 reflects confounding by socio-demographic variables included in the multivariable model.

• Specifically, factors such as age, educational attainment, and socioeconomic status partially accounted for the crude association, indicating that these variables are important determinants of both ACE exposure and multimorbidity.

• Where analytically feasible, we have indicated the covariate(s) that contributed most to the reduction in the effect estimate, thereby providing a clearer interpretation of the independent association between ACE exposure and multimorbidity.

• This additional discussion strengthens the causal interpretability of our findings while maintaining appropriate caution.

• (page 10, line 314 to 316).

5. Inclusion of Methodological Details as Supplementary Material

In line with your recommendation, we have included a comprehensive description of the study methodology, rather than referring readers to prior publications alone. This includes:

• Sampling procedures

• Measurement of ACEs and multimorbidity

• Data collection protocols

• Analytical framework

(Pages 3 to 4, lines 174 to 189)

This addition enhances the manuscript’s transparency and allows for independent appraisal of the study design and execution.

In summary, we have carefully addressed all comments and believe that the revisions have substantially improved the clarity, methodological robustness, and interpretative balance of the manuscript. We are grateful for the opportunity to refine our work and for the valuable guidance provided.

Thank you for your consideration.

Yours sincerely,

Nestor Asiamah

On behalf of all authors

Attachment Submitted filename: Authors Response.docx

# Editor report: Decision Letter 3

**DOI:** 10.1371/journal.pone.0358944.r007

**Authors:** Mithun Sikdar

9 Sep 2026

The Impact of Adverse Childhood Experiences on the Development of Multimorbidity in Older Adults in Nigeria

PONE-D-25-20171R3

Dear Dr.Nestor Asiamah,

We’re pleased to inform you that your manuscript has been judged scientifically and it is now suitable for publication. It will be formally accepted for publication once it meets all outstanding technical requirements.

Within one week, you’ll receive an e-mail detailing the required amendments. When these have been addressed, you’ll receive a formal acceptance letter and your manuscript will be scheduled for publication.

An invoice will be generated when your article is formally accepted. Please note, if your institution has a publishing partnership with PLOS and your article meets the relevant criteria, all or part of your publication costs will be covered. Please make sure your user information is up-to-date by logging into Editorial Manager at Editorial Manager® and clicking the ‘Update My Information' link at the top of the page. For questions related to billing, please contact billing support.

If your institution or institutions have a press office, please notify them about your upcoming paper to help maximize its impact. If they’ll be preparing press materials, please inform our press team as soon as possible -- no later than 48 hours after receiving the formal acceptance. Your manuscript will remain under strict press embargo until 2 pm Eastern Time on the date of publication. For more information, please contact onepress@plos.org.

As a minor editorial point, I suggest that you include a brief statement at the end of the manuscript regarding the use of any AI-based tools during manuscript preparation, including language editing, proofreading, or other forms of editorial assistance, if applicable.

Kind regards,

Mithun Sikdar

Academic Editor

PLOS One

# Editor report: Acceptance letter

**DOI:** 10.1371/journal.pone.0358944.r008

**Authors:** Mithun Sikdar

PONE-D-25-20171R3

PLOS One

Dear Dr. Asiamah,

I'm pleased to inform you that your manuscript has been deemed suitable for publication in PLOS One. Congratulations! Your manuscript is now being handed over to our production team.

At this stage, our production department will prepare your paper for publication. This includes ensuring the following:

* All references, tables, and figures are properly cited

* All relevant supporting information is included in the manuscript submission,

* There are no issues that prevent the paper from being properly typeset

You will receive further instructions from the production team, including instructions on how to review your proof when it is ready. Please keep in mind that we are working through a large volume of accepted articles, so please give us a few days to review your paper and let you know the next and final steps.

Lastly, if your institution or institutions have a press office, please let them know about your upcoming paper now to help maximize its impact. If they'll be preparing press materials, please inform our press team within the next 48 hours. Your manuscript will remain under strict press embargo until 2 pm Eastern Time on the date of publication. For more information, please contact onepress@plos.org.

You will receive an invoice from PLOS for your publication fee after your manuscript has reached the completed accept phase. If you receive an email requesting payment before acceptance or for any other service, this may be a phishing scheme. Learn how to identify phishing emails and protect your accounts at https://explore.plos.org/phishing.

If we can help with anything else, please email us at customercare@plos.org.

Thank you for submitting your work to PLOS One and supporting open access.

Kind regards,

PLOS One Editorial Office Staff

on behalf of

Dr. Mithun Sikdar

Academic Editor

PLOS One
